# Decoding the base flipping mechanism of the SET- and RING-associated (SRA) domain of the epigenetic UHRF1 protein

**DOI:** 10.1093/nar/gkaf909

**Published:** 2025-09-17

**Authors:** Dipanjan Mukherjee, Stefano Ciaco, Lara Martinez-Fernandez, Krishna Gavvala, Elisa Bombarda, Aurélie Bourdérioux, Dmytro Dziuba, Fabien Hanser, Nicolas Humbert, Aqib Javed, Marc Mousli, Pankhi Singh, Yitzhak Tor, Roberto Improta, Mattia Mori, Yves Mély

**Affiliations:** Laboratoire de Bioimagerie et Pathologies, UMR 7021 CNRS Université de Strasbourg, Faculté de pharmacie, 74 route du Rhin, Illkirch 67401, France; Laboratoire de Bioimagerie et Pathologies, UMR 7021 CNRS Université de Strasbourg, Faculté de pharmacie, 74 route du Rhin, Illkirch 67401, France; Department of Biotechnology, Chemistry and Pharmacy, University of Siena, Siena 53100, Italy; Departamento de Química Física de Materiales, Instituto de Química Física Blas Cabrera, CSIC (Consejo Superior de Investigaciones Científicas), Madrid 28006, Spain; Laboratoire de Bioimagerie et Pathologies, UMR 7021 CNRS Université de Strasbourg, Faculté de pharmacie, 74 route du Rhin, Illkirch 67401, France; Laboratoire de Bioimagerie et Pathologies, UMR 7021 CNRS Université de Strasbourg, Faculté de pharmacie, 74 route du Rhin, Illkirch 67401, France; Laboratoire de Bioimagerie et Pathologies, UMR 7021 CNRS Université de Strasbourg, Faculté de pharmacie, 74 route du Rhin, Illkirch 67401, France; Laboratoire de Bioimagerie et Pathologies, UMR 7021 CNRS Université de Strasbourg, Faculté de pharmacie, 74 route du Rhin, Illkirch 67401, France; Laboratoire de Bioimagerie et Pathologies, UMR 7021 CNRS Université de Strasbourg, Faculté de pharmacie, 74 route du Rhin, Illkirch 67401, France; Laboratoire de Bioimagerie et Pathologies, UMR 7021 CNRS Université de Strasbourg, Faculté de pharmacie, 74 route du Rhin, Illkirch 67401, France; Laboratoire de Bioimagerie et Pathologies, UMR 7021 CNRS Université de Strasbourg, Faculté de pharmacie, 74 route du Rhin, Illkirch 67401, France; Laboratoire de Bioimagerie et Pathologies, UMR 7021 CNRS Université de Strasbourg, Faculté de pharmacie, 74 route du Rhin, Illkirch 67401, France; Laboratoire de Bioimagerie et Pathologies, UMR 7021 CNRS Université de Strasbourg, Faculté de pharmacie, 74 route du Rhin, Illkirch 67401, France; Department of Chemistry and Biochemistry, University of California, San Diego, La Jolla, CA 92093-0358, United States; Consiglio Nazionale delle Ricerche, Istituto Biostrutture e Bioimmagini, Via De Amicis 95, 80145 Napoli, Italy; Department of Biotechnology, Chemistry and Pharmacy, University of Siena, Siena 53100, Italy; Laboratoire de Bioimagerie et Pathologies, UMR 7021 CNRS Université de Strasbourg, Faculté de pharmacie, 74 route du Rhin, Illkirch 67401, France

## Abstract

Ubiquitin-like, containing PHD and RING fingers domains 1 (UHRF1) plays a pivotal role in replicating DNA methylation patterns during cell division. Acting as a DNA reader, UHRF1, via its SET- and RING-associated (SRA) domain, recognizes hemi-methylated (HM) CpG sites and flips 5-methylcytosine (5mC) nucleobases. This flipping triggers DNA methyltransferase 1 (DNMT1) recruitment to methylate cytosine in the complementary strand. To investigate the SRA-induced base-flipping mechanism, we introduced thienoguanosine (^th^G), a fluorescent guanosine analogue, at four positions in HM and non-methylated duplexes. The interactions of these labelled duplexes with wild-type SRA and a G448D mutant (incapable of base-flipping) were monitored using a combination of stopped-flow fluorescence measurements, molecular dynamics simulations, and quantum mechanical calculations. We show that 5mC and C residues are flipped with similar rate constants. However, while C residues rapidly revert to their original state, enabling SRA to continue reading or dissociate, SRA complexes with flipped 5mC undergo a slow conformational rearrangement, leading to the final conformation crucial for DNMT1 recruitment. Taken together, our findings suggest that base flipping is used to discriminate 5mC from C residues, while the ensuing conformational rearrangement drives DNMT1 recruitment.

## Graphical Abstract

**Graphical Abstract:**
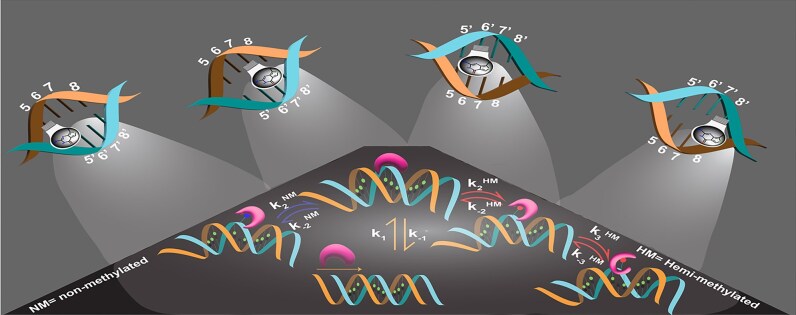

## Introduction

Covalent modifications of DNA nucleobases, such as cytosine methylation [1–5], are crucial for epigenetic regulation. In mammalian cells, 70%–80% of cytosines are methylated at position 5 in mCpG sites [6–10]. This methylation regulates gene expression, imprinting, and stability, playing a key role in cell development, differentiation, and X chromosome inactivation [6, 11–14]. To preserve cell identity, the methylation patterns need to be faithfully copied during cell divisions. This is ensured by a multi-protein DNA methylation machinery, in which DNA methyltransferase 1 (DNMT1) and Ubiquitin-like, containing PHD and RING fingers domains 1 (UHRF1) are two main actors [2, 15, 16]. During DNA replication, UHRF1 binds to hemi-methylated DNA (HM DNA), preferentially recognizing mCpG sites [17–19] and flipping out the 5-methylcytosine (5mC) nucleobases through its SET- and RING-associated (SRA) domain. This base flipping facilitates the recruitment of DNMT1, which in turn flips out the opposite non-methylated (NM) cytosine (C) of the complementary strand and methylates it [15–18, 20–23]. The flipping of 5mC by SRA thus plays a pivotal role in the faithful replication of DNA methylation patterns.

**Figure 1.**
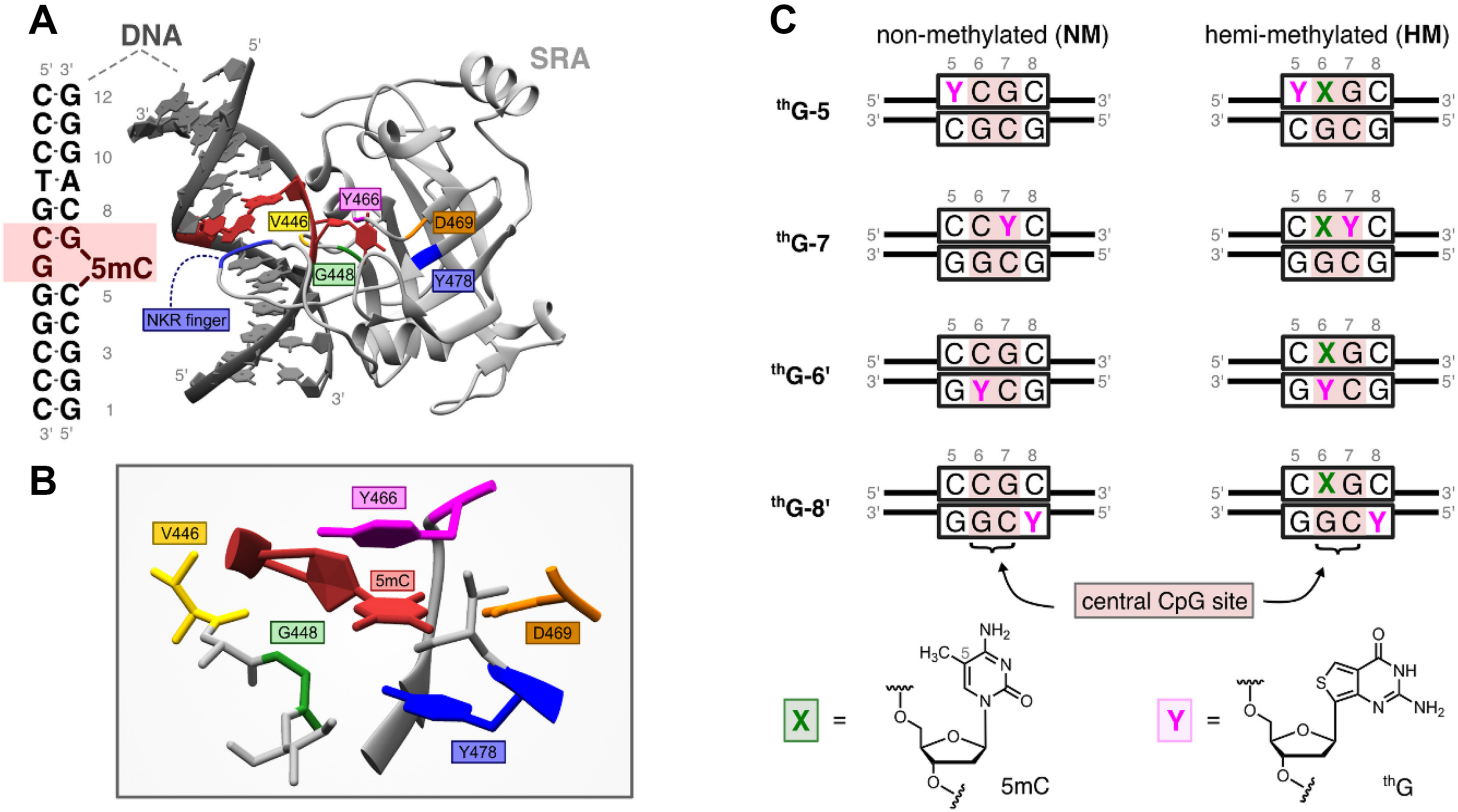
(**A**) Sequence of the HM dodecamer DNA duplex used in this study and cartoon representation of the SRA-induced base flipping of 5mC from the central mCpG site (highlighted in red). Key amino acid residues involved in the stabilization of the base-flipped conformation are indicated. (**B**) Close-up view of the 5mC-binding pocket of the SRA domain, highlighting key amino acid residues involved in the base flipping process. Both structural cartoons were generated from the crystal structure with PDB ID 3CLZ [19] using UCSF Chimera [40]. (**C**) HM and NM fluorescently labeled duplexes used in this study. Four distinct positions (5, 7, 6′, and 8′) of the duplex sequence shown in panel A (left) were individually substituted with thienoguanosine (^th^G), denoted by Y; 5mC is denoted by X. At position 7, 6′, and 8′, ^th^G replaced a G residue while at position 5, ^th^G replaced a C residue. In the latter case, we simultaneously replaced the opposite G at position 5′ by a C residue to preserve the overall base complementarity and stability of the duplex.

A critical advancement in understanding the base flipping process has been afforded by the X-ray crystal structures of the SRA domain from human or mouse UHRF1 protein in complex with short HM duplexes [17–19]. The SRA domain resembles a hand grasping the HM duplex in its palm, while its thumb (Val 446 in human SRA) and NKR finger (Asn 489, Lys 490, and Arg 491 in human SRA) are crucial in flipping and stabilizing the 5mC nucleobase in the SRA-binding pocket (Fig. 1A). Although highly useful in revealing the detailed structure of the final SRA–DNA complex, the crystallographic data do not provide any information about the discrete steps leading to this final complex and their kinetics. To decipher the mechanism of base-flipping processes, fluorescence-based stopped-flow techniques using nucleic acids labelled with fluorescent nucleoside analogues (FNAs) have proved to be highly useful. By primarily using 2-aminopurine (2-AP), a FNA of adenosine, the base flipping mechanisms of several enzymatic proteins, such as formamidopyrimidine-DNA glycosylase (FPG), uracil DNA glycosylase (UDG), EcoRI DNA methyltransferase (M.EcoRI), *Escherichia coli* Dam DNA-(adenine-N6)-methyltransferase (Ecodam), *E. coli* 3-methyladenine DNA glycosylase II (*E. coli* Alka), Human alkyladenine DNA glycosylase (AAG), DNA cytosine-5-methyltransferase HhaI (M.HhaI), *E. coli* Endonuclease VIII (Endo VIII), and *E. coli* MutY adenine glycosylase (MutY) have been resolved [24–39]. For most systems, base flipping was found to follow a diffusion-limited non-specific binding and sliding of the enzyme onto the nucleic acid sequence and to precede a slower conformational step that leads to the enzymatic reaction.

In contrast to these enzymes, a former study with the non-enzymatic SRA domain of UHRF1 indicated that 2-AP inserted at various positions next to the CpG-binding site was not suitable for monitoring the SRA-induced base flipping process [41], due to its low sensitivity and inability to discriminate SRA activity on HM and NM duplexes. Much more promising results were instead obtained with thienoguanosine (^th^G), a FNA that nearly perfectly replaces guanosine (G) in nucleic acids [42–44]. Indeed, replacing the natural nucleobase G, adjacent to the target 5mC, by ^th^G in the mCpG site was found to preserve the SRA binding preference to HM duplexes over NM duplexes [45], while a 2-fold larger fluorescence increase was observed for SRA interacting with ^th^G-labelled HM duplexes as compared to NM duplexes [45]. Our initial interpretation was that the fluorescence increase seen for NM duplexes was due to ^th^G environmental change induced by SRA binding to the CpG site, while the additional fluorescence increase seen with HM duplexes was due to the flipping of 5mC, resulting in a loss of base stacking with the emissive G analogue. Based on this interpretation, we proposed a two-step mechanism where a fast binding step was followed by a slower base flipping step [45]. However, our more recent study has shown that binding of a ^th^G-labelled HM duplex by the SRA G448D mutant, which is unable to flip the 5mC base due to steric hindrance introduced by the Asp residue at position 448 in the binding pocket [18], induced only minimal change in fluorescence [46], clearly challenging our previously proposed model.

In this report, our aim was to perform an in-depth kinetic investigation of the SRA-induced base flipping mechanism, by combining stopped-flow experiments with molecular dynamics simulations (MD) and quantum mechanical (QM) calculations. To this end, we selected the 12-bp duplex sequence, whose crystallographic structure with SRA has been previously reported [18] (Fig. 1A and B), and replaced the natural nucleobases by ^th^G at four positions within or immediately adjacent to the CpG site (Fig. 1C). Taken together, our data reveal that SRA-induced base flipping occurs in both HM and NM duplexes. However, C rapidly flips back in the NM duplex, whereas a slow conformational rearrangement stabilizes the SRA/HM complex with the 5mC flipped into the SRA.

## Materials and methods

### ^th^G-labelled oligonucleotides

2′-Deoxy-D-ribosyl-thienoguanosine (^th^G) and its phosphoramidite were synthesized as described [43, 44]. The ^th^G-labelled sequences (Fig. 1C) were synthesized and reverse phase HPLC-purified by TriLink Biotechnologies (USA) and Eurogentec (Belgium), while the unlabelled strands were obtained from Eurogentec and IBA GmbH. To form the duplexes, the ^th^G-labelled oligonucleotides at 100 μM were mixed with their complementary unlabelled oligonucleotides at 110 μM in a 20 mM PBS buffer pH 7.5, 150 mM NaCl. The duplexes were annealed using a polymerase chain reaction (PCR) machine (Bio-Rad, USA), starting with an initial heating step at 95°C for 5 min, followed by gradual cooling to room temperature over 2 h. The resulting duplexes were stored as small aliquots at –20°C before use. Each labelled duplex is identified through its ^th^G position (^th^G 7, ^th^G 5, ^th^G 6′, and ^th^G 8′) and its methylation status (NM and HM) (Fig. 1C).

### Expression and purification of SRA and SRA G448D

**Figure 2.**
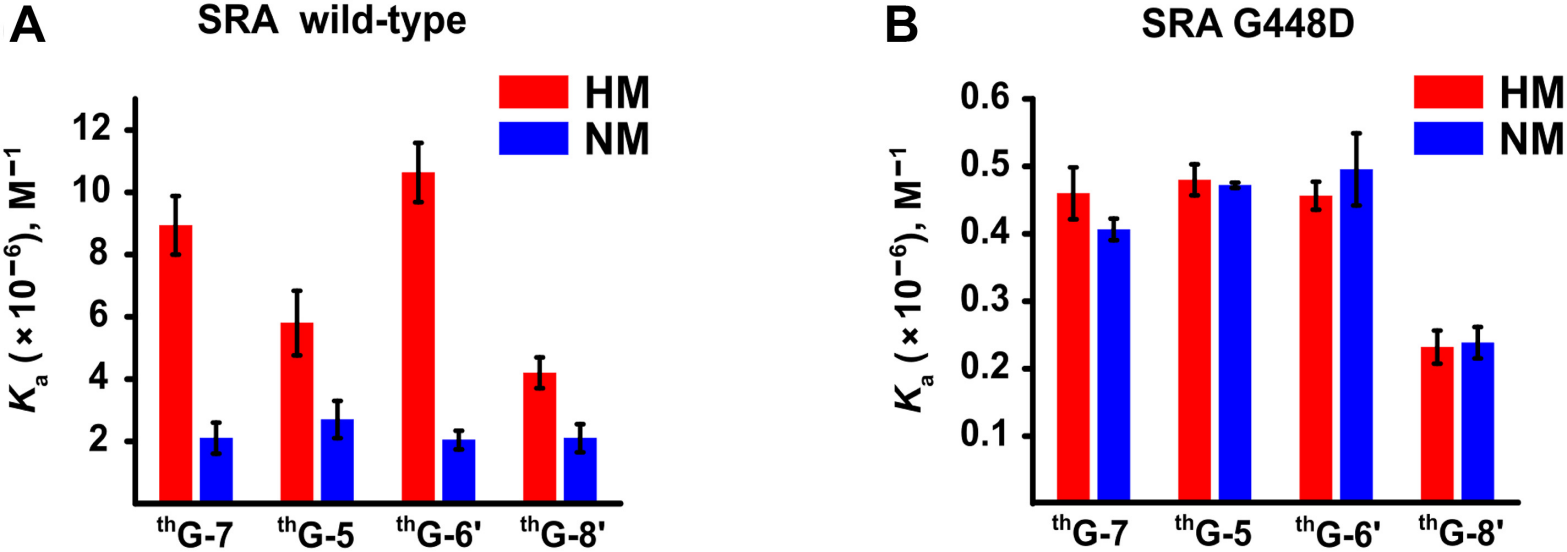
Association constants (*K*_a_) of the ^th^G-labelled HM and NM duplexes to (**A**) SRA and (**B**) SRA G448D, determined by fluorescence anisotropy titrations (Supplementary Figs S2 and S3). Duplex concentration was 1 μM. Binding constants were determined by fitting the anisotropy curves to equations 1 and 2, using a binding stoichiometry of 1. When the number of binding sites was left floating, values close to 1 were systematically obtained (data not shown). All experiments were carried out in 20 mM PBS pH 7.5, 50 mM NaCl, 2.5 mM TCEP, and 0.05% PEG 20000 at 20°C. Data are presented as means and standard deviations for *n* = 3 replicates.

**Figure 3.**
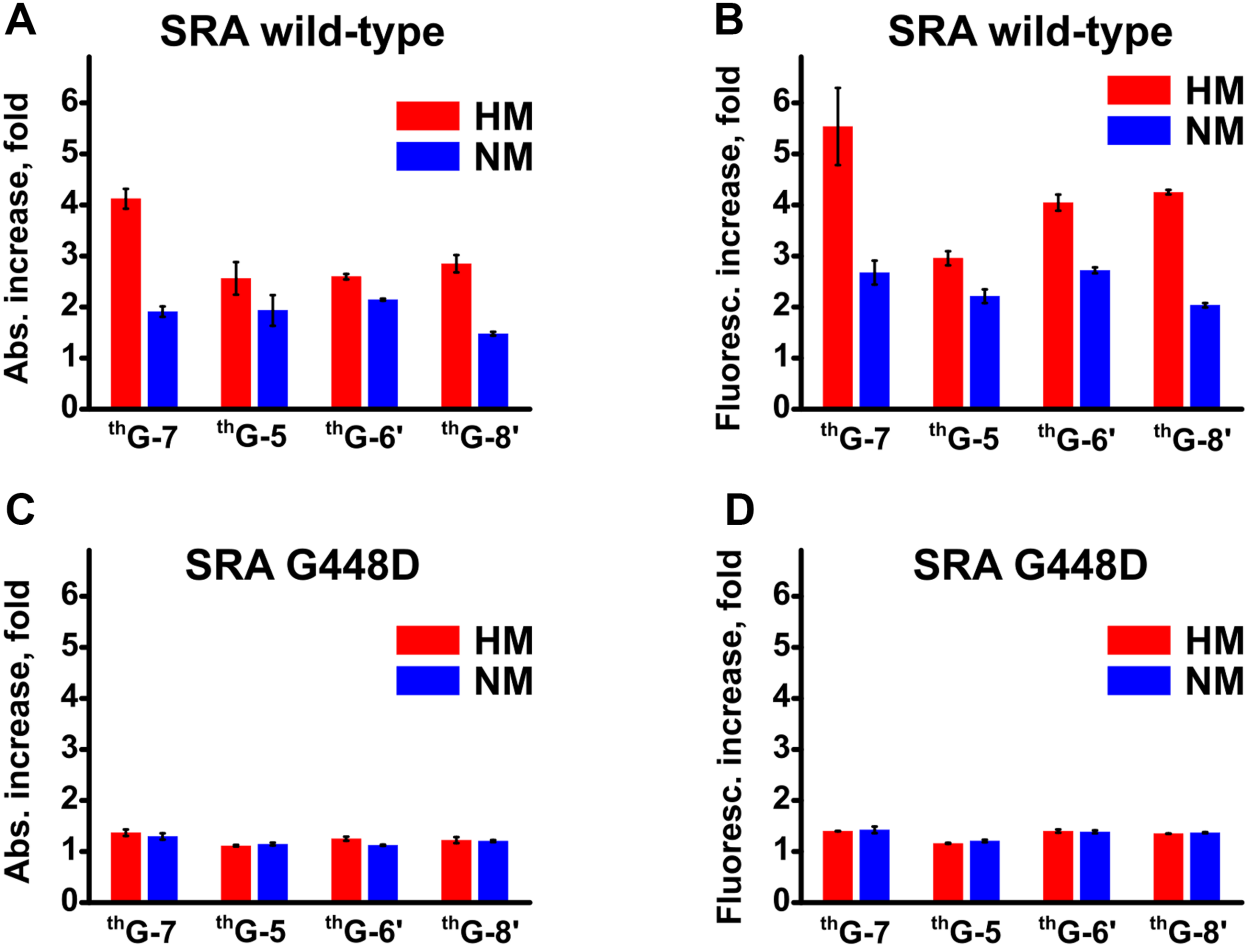
Increase in the absorption and fluorescence of ^th^G-labelled HM and NM duplexes upon addition of SRA (**A** and **B**) or SRA G448D (**C** and **D**). The increase in absorbance was calculated as *A*_SRA_/*A*_free_, where *A*_SRA_ and *A*_free_ are the integrals of the deconvoluted ^th^G ππ* absorption band of the ^th^G-labelled duplexes, in the presence and absence of SRA, respectively (panels A–D in Supplementary Figs S6 and S7). The increase in fluorescence was calculated as *F*_SRA_/*F*_free_, where *F*_SRA_ and *F*_free_ are the integrals of the fluorescence spectra in the presence and absence of SRA, respectively (panels E–H in Supplementary Figs S6 and S7). Excitation wavelength was 350 nm. Experimental conditions are as in Fig. 2. Data are presented as means and standard deviations for *n* = 3–5 replicates.

The SRA domain (residues 408−643 of human UHRF1) and its mutant in which the glycine at position 448 is substituted by an aspartic acid (SRA G448D) were expressed in *E. coli* BL21(DE3)pLysS and purified according to a previously described protocol with several modifications [47]. Instead of LB (Luria Broth), TB media (Terrific Broth) was used, and isopropylthio-β-galactoside (IPTG) was added when the absorbance at 600 nm reached between 0.45 and 0.50. The benefits of using TB media for preparing recombinant proteins have been well documented [48]. Moreover, to minimize protein degradation and preserve activity, all purification steps were performed at 4°C, with the entire process from cell lysis to flash freezing of purified proteins, completed within one day. This modified strategy led to (i) a 3-fold increase in the yield of protein and (ii) an improved and reproducible protein activity, with ∼40% signal increase in base flipping assays compared to SRA prepared using the previous protocol [45–47]. The molecular mass of SRA and SRA G448D was confirmed to be ∼ 34 kDa, using 10% Sodium Dodecyl Sulfate PolyAcrylamide Gel Electrophoresis (SDS−PAGE) (Supplementary Fig. S1) and the Color Prestained Protein Standard, Broad Range (10–250 kDa) ladder from New England Biolabs (catalog no. P7719S). SRA activity after production of each batch was tested using the ^th^G 7-labelled HM duplex (Fig. 1C) by measuring the SRA binding constant (Fig. 2) and SRA-induced fluorescence increase (Fig. 3). The prepared SRA is validated when its binding constant is within 20% of its target value (*K*_a_= 8 × 10^6^ M^–1^, [45]) and it increases the emission of the ^th^G 7-labelled HM duplex by 5- to 6-fold [46]. For SRA G448D, the preparation is validated when its binding constant is within 20% of its target value (*K*_a_= 5 × 10^5^ M^−1^, [45]).

### UV-visible absorption

The absorption spectra were recorded with a Cary 4000 spectrophotometer (Varian). The concentrations of the ^th^G-labelled and unlabelled oligonucleotides were determined using the molar absorption coefficients at 260 nm, ϵ_260_, provided by the suppliers (ϵ_unlabelled duplex_= 186 300 M^–1^s^–1^, ϵ^th^G 7 = 183 300 M^–1^s^–1^, ϵ^th^G 5 = 180 100 M^–1^s^–1^, ϵ^th^G 6’= 183 300 M^–1^s^–1^, ϵ^th^G 8’= 183 300 M^–1^s^–1^). A molar absorption coefficient of 43 890 M^–1^ cm^–1^ at 280 nm was used to determine the concentration of SRA or SRA G448D [45, 46]. The spectra were recorded in 20 mM PBS buffer pH 7.5, 50 mM NaCl, 2.5 mM TCEP, and PEG20000 (0.05%) used to prevent protein adsorption onto the cuvette walls [45]. To get accurate measurements of the ^th^G absorption band, the spectra of the ^th^G-labelled duplexes were recorded at 10 μM. The complexes were prepared by combining 12 μM SRA or 20 μM SRA G448D with 10 μM duplexes (final concentrations) to ensure that >80% of the duplexes are bound by the proteins. Deconvolution of the absorption spectra of the ^th^G-labelled oligonucleotides to obtain the ππ* absorption band was carried out as previously described [46].

### Steady-state fluorescence spectroscopy and anisotropy

Fluorescence spectra of ^th^G-labelled duplexes were recorded with a Horiba Fluorolog fluorimeter, equipped with a Peltier thermostat to maintain the cell temperature at 20°C. The fluorescence spectra were corrected from buffer background emission, lamp fluctuations and wavelength dependence of the set-up.

Steady-state fluorescence anisotropy titrations of ^th^G-labelled duplexes at 1 μM with increasing SRA concentrations have been performed using a Horiba Fluorolog fluorimeter in T-format. Fluorescence anisotropy is monitored at 454 nm by exciting at 350 nm. During the titration, for each SRA concentration, the anisotropy value corresponds to the average calculated over 10 measurements and taking into account the G-factor correction [49]. From the titration, the equilibrium association constant *K*_a_ of SRA or SRA G448D to the labelled duplexes is determined by equations (1) and (2):


(1) \begin{eqnarray*}
r = \frac{{\vartheta \times R \times {r_t} - {r_d} \times \left( {\vartheta - 1} \right)}}{{1 + R \times \vartheta - \vartheta }}
\end{eqnarray*}

where *r* and *r_t_* are, respectively, the anisotropy of ^th^G-labelled duplexes for a given protein concentration and at saturation, *r*_d_ is the anisotropy in absence of protein, *R* is the ratio of the quantum yield of the bound to the free duplex which therefore takes into account the changes in fluorescence intensity during the titration, and $\vartheta$ is the fraction of protein bound to the duplexes, calculated by:


(2) \begin{eqnarray*}
\vartheta = \frac{{\left( {K_a^{ - 1} + n \cdot {N_t} + {P_t}} \right) - \sqrt {{{\left( {K_a^{ - 1} + n \cdot {N_t} + {P_t}} \right)}^2} - 4n \cdot {P_t} \cdot {N_t}} }}{{2{N_t}}}
\end{eqnarray*}

where *P*_t_ and *N*_t_ are the total concentrations of protein and ^th^G-labelled duplexes, respectively, and*n* is the number of proteins bound per duplex and considered to be 1.

### Stopped-flow kinetics

The stopped-flow kinetics of ^th^G-labelled HM and NM duplexes interacting with SRA and SRA G448D have been investigated at 20°C using a SFM-3 setup (Biologic, France). We set the excitation wavelength at 365 nm and measured the emission intensity above 420 nm using a long-pass filter (ITOS GmbH, Germany). The high voltage (HV) is maintained at 580 V for a good signal-to-noise ratio. Specifically, for the NM duplexes, the reaction is completed within 0.1 s. Two collection regimes for the data points were used. In the first, we collected the maximum number of data points (10 000) within 0.1 s, and the remaining 9000 data points were recorded between 0.1 and 1 s. In the second regime, 2000 points were collected in the first 0.1 s and 9000 points over the remaining 0.9 s. Then, the data points have been further averaged by group of 4 (Origin: Worksheet > Reduce by Group > *N* = 4). The two approaches were checked to provide the same *k*_obs_ values, but the second approach significantly improved the visualization of the early kinetic phases. The dead time for our setup under these experimental conditions was 2.7 ms. To capture all the kinetic changes of the fast phase, we programmed the PMT detector activation for 40 ms before mixing the solution in the chamber, and special care was taken to degas the buffer. The apparent rate constants *k*_obs_ and the amplitudes were determined from the kinetic traces by extrapolating them to the true time zero by taking in account the dead time. The oligonucleotide concentration after mixing was 0.3 μM, and the protein was added at a concentration varying from 1.5 to 9 μM.

Dissociation experiments were carried out using an excess of calf thymus DNA as a competitor. Calf thymus DNA (Type I fibers, sodium salt; Sigma-Aldrich, catalog no. D1501) was dissolved in PBS buffer (pH 7.5) and stirred overnight to ensure complete solubilization. The concentration of the stock solution expressed in nucleotides was determined by UV–Vis spectroscopy using an extinction coefficient ϵ = 6 600 M^-1^ cm^-1^. Complexes were pre-formed by incubating 0.6 μM of HM or NM duplexes with either 1.5 μM SRA (for HM duplexes) or 3.0 μM SRA (for NM duplexes), ensuring substantial complex formation based on their respective binding affinities. The pre-formed complex was loaded into one syringe of the stopped-flow apparatus, while the second syringe contained 1500 μM calf thymus DNA. Upon mixing of equal volumes of the two syringes, the final concentration of calf thymus DNA in the reaction mixture was 750 μM. Dissociation of the complexes of SRA with NM and HM duplexes was monitored by observing the decrease in ^th^G fluorescence intensity. All measurements were carried out under the same buffer conditions and data acquisition settings as described above, including the instrument’s dead time. All time-resolved kinetic phases with HM duplexes were analysed using a bi-exponential function.


(3) \begin{eqnarray*}
y = {I_{\rm f}} - \left( {{I_{\rm f}} - {I_{\rm i}}} \right)\left[ {a \cdot {e^{ - {k_{obs1}} \cdot t}} + \left( {1 - a} \right) \cdot {e^{ - {k_{obs2}} \cdot t}}} \right]
\end{eqnarray*}

Here, *I*_f_ and *I*_i_ represent the final and initial fluorescence intensity values, respectively, while *k*_obs1_ and *k*_obs2_ are the observed rate constants for the fast and slow resolvable phases, respectively. The amplitude of the fast kinetic phase is denoted by $a$. With NM duplexes, we reduced equation (3) to a monoexponential equation $( {a = 1} )$ in order to fit the data and extract the *k*_obs1_ value. For the dissociation experiments—regardless of whether HM or NM duplexes were used—the fluorescence decay traces were fitted to a single-exponential function using equation (3), with the parameter *a* fixed at 1. All fittings were carried out with the Origin (Microcal, Northampton, MA) software based on the non-linear least-square method applying the Levenberg–Marquardt algorithm.

### Numerical analysis with Dynafit Software

To validate the proposed kinetic models and determine the associated rate constants, the experimental traces were further analysed using the numerical solving software Dynafit [50]. This software performs least-squares fits of the kinetic traces using the classical Levenberg–Marquardt algorithm. Since the intensities of the kinetic traces are not corrected for lamp fluctuations and instrumental responses, we rescaled the plateau values of the kinetic curves according to the binding parameters and steady-state fluorescence increases shown in Figs 2 and 3, respectively. The response values of the free duplexes in Dynafit were obtained by dividing the measured intensity values by the concentration of the free duplex (0.3 μM). The response values of the other species involved in the reaction mechanism were expressed as a function of the response value of the free duplex.

### MD simulation and QM calculations

MD simulations were run with AMBER 18 using ^th^G parameters that have been previously validated, and a consolidated MD protocol [47, 51–55]. Macromolecular assemblies were built on the X-ray crystallography complex of SRA in complex with the 12 bp HM duplex (PDB ID: 3CLZ) [18] (Fig. 1A). Replacement of G7 with ^th^G and single point mutations (i.e. G448D) were introduced with the LEaP program [56]. The SRA-free duplex was built in Avogadro version 1.2.0 [56, 57], while 5mC parameters were retrieved from the Amber Parameter Database (APD, http://amber.manchester.ac.uk) [58]. In all MD simulations, the ff14SB force field was used to parametrize SRA, and the OL15 force field was used to parametrize the DNA duplex [59, 60]. Macromolecular systems were solvated in a rectilinear box of TIP3P type water molecules buffering 14 Å, while Na^+^ ions were added up to charge neutrality. In all simulations, a 2 fs time-step was used. Each solvated system was first energy minimized through a 2-run procedure consisting in (i) energy minimization of the solvent for 1500 steps with the steepest descent algorithm (SD) followed by 3500 steps with the conjugate gradient algorithm (CG), and (ii) energy minimization of the solvated solute for 1500 steps with the SD followed by 6000 steps with the CG. Energy minimized systems were then heated to 300 K with the Langevin thermostat at constant volume for 2 ns; density was equilibrated for 2 ns at constant pressure with the Berendsen barostat, and the systems were then relaxed through a preliminary MD run of 50 ns. Finally, unrestrained MD trajectories were run at constant pressure for 500 ns. MD analysis was carried out with the CPPTRAJ software [61]. Graphics are generated with an open source build of PyMOL, version 2.2.0 [62].

For QM calculations, our computational model included 5 or 6 bases of the ^th^G-labelled HM duplex (namely residues 6, 7, 8, and the complementary bases) considering also the phosphodeoxy-ribose backbone, at the fully QM level. The geometries of the ground electronic state have been optimized with the Density Functional Theory (DFT) method using the M052X [63, 64] functional combined with the 6-31G(d) basis set, considering solvation via an implicit model (Polarizable Continuum Model, PCM [65, 66]). Excited state energies and intensities (oscillator strength) have been computed on top of these geometries at the same level of theory but resorting to the Time-Dependent version of DFT (TD-DFT). This approach has been selected due to its good performance in previous studies on ^th^G fluorescence properties both in solution and within duplexes [46, 52]. All calculations were carried out with Gaussian16 [67]. Finally, as detailed below, to obtain further information on the effect of the duplex geometry on ^th^G photophysics, we repeated our analysis using two representative structures from the MD simulations as starting geometries.

## Results

### Binding constants of ^th^G-labelled oligonucleotides for SRA and SRA G448D

Firstly, to test the impact of the replacement of the natural nucleobases by ^th^G, we systematically determined the binding constants of the labelled NM and HM duplexes for SRA (Fig. 2A) and SRA G448D (Fig. 2B) by performing fluorescence anisotropy titrations (Supplementary Figs S2 and S3). For all labelled HM duplexes, the affinity for SRA (Fig. 2A) differed by a factor of less than two as compared to the affinity for the non-labelled HM duplex determined by isothermal calorimetry [5.3 (±0.7) × 10^6^ M^–1^] [45] or by monitoring SRA intrinsic fluorescence [8.1 (±0.2) × 10^6^ M^–1^, Supplementary Fig. S4]. This difference in binding energy corresponds to a free energy difference of <450 cal/mol, which is substantially less than the free energy of a hydrogen bond (1–5 kcal/mole) and indicates that the impact of ^th^G is minimal. For the corresponding ^th^G-labelled NM duplexes, the values of SRA affinity are in line with that for the non-labelled duplexes [1.43 (±0.07) × 10^6^ M^–1^, Supplementary Fig. S4] and consistently 2- to 4-fold lower than for HM duplexes, indicating that ^th^G preserved the SRA binding preference for HM duplexes [16–19, 68–71] and therefore nearly perfectly replaced the natural nucleobases. Of note, the modest binding preference of SRA for HM duplexes was confirmed by comparison with non-CpG-containing duplexes, showing affinities comparable to NM duplexes (data not shown), in line with previous studies [41, 71–73] and with the reader role of UHRF1. Indeed, if SRA affinity for non-CpG sites would be too low, the protein would rapidly dissociate and terminate the reading process. Similarly, if the affinity of mCpG sites was too high, the protein would be blocked at this site, which would again impact the reading process.

In contrast, the binding constants of SRA G448D (∼2.5–5 × 10^5^ M^−1^) were similar for HM and NM duplexes, irrespective of the labelled position, and one order of magnitude lower than those of SRA (Fig. 2B). Thus, replacement of G448 by D is confirmed to diminish affinity and abolish preference for HM duplexes [18, 45], independently on the position of ^th^G in the sequence. Moreover, similar binding constants were obtained with duplexes where the CpG motifs were replaced by CpA or CpT motifs, indicating that SRA G448D binding is essentially non-specific (Supplementary Fig. S5).

### Changes in absorption and emission spectra of ^th^G-labelled duplexes on interaction with SRA and SRA G448D

We previously showed that in the free HM duplex labelled at position 7, the ππ* absorption band responsible for ^th^G emission coexists with a non-emissive blue-shifted band resulting from charge transfer (CT) between ^th^G and its flanking residues [46]. Moreover, we showed that base flipping of 5mC at position 6 by SRA strongly increases this ππ* absorption band, providing a nearly parallel increase in ^th^G emission. These findings indicated that the major contribution to the fluorescence enhancement originates from an increase in absorbance, rather than from a substantial change in fluorescence quantum yield. In this context, our aim was to investigate if similar spectral changes can be observed when ^th^G is incorporated at position 5, 6′, and 8′ in HM and NM duplexes.

For all four positions, addition of SRA to the labelled HM duplexes induced a large increase in ^th^G’s ππ* absorption (Fig. 3A, red bars and Supplementary Fig. S6, panels A–D) and emission (Fig. 3B, red bars and Supplementary Fig. S6, panels E–H), indicating that all four positions can be used to monitor the DNA–protein interaction. The largest changes (4- and 5.5-fold, respectively) were observed for ^th^G at position 7 (Fig. 3A and B, red bars), which is thus the most sensitive position to SRA interaction. For the other three positions (5, 6′, and 8′), the changes in ππ* absorption and emission were 2- to 3-fold and 3- to 4-fold, respectively, suggesting that structural perturbations are not restricted to the immediate vicinity of 5mC.

Interestingly, a 2- to 2.7-fold increase in both ^th^G absorption and emission was also observed when SRA was added to the NM duplexes (Fig. 3A and B, blue bars). Comparison of the red and blue bars in Fig. 3A and B indicates that the fluorescence and absorption increases for NM duplexes are systematically 1.3- to 2-fold lower than for HM duplexes, suggesting that SRA induced smaller changes in NM duplexes as compared to HM duplexes at all positions.

SRA G488D used at a concentration ensuring at least 80% duplex binding, induced only modest changes (on the average 1.2- to 1.3-fold) in the absorption and emission of ^th^G, independently of its position and the methylation state of the C residue at position 6 (Fig. 3C and D, Supplementary Fig. S6A–H and Supplementary Fig. S7A–H). This confirms that SRA G448D interacts in the same manner with HM and NM duplexes, likely inducing only marginal structural changes in the bound duplex.

### Kinetics of SRA and SRA G448D interaction with NM and HM duplexes

**Figure 4.**
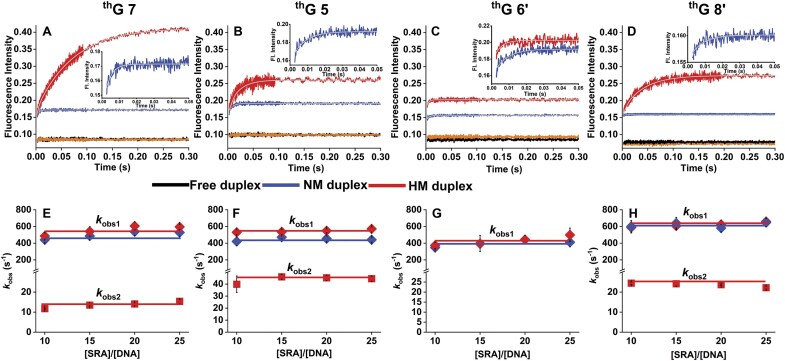
Interaction kinetics of SRA with HM and NM duplexes labeled with ^th^G at positions 7 (**A** and **E**), 5 (**B** and **F**), 6′ (**C** and **G**), and 8′ (**D** and **H**), measured by stopped-flow fluorescence. The concentration of each duplex was 0.3 μM after mixing. Excitation was at 365 nm, and emission was collected above 420 nm using a long-pass filter. Experiments were carried out in the same buffer as in Fig. 2. Panels A–D show representative kinetic traces. Control traces obtained by mixing HM and NM duplexes with buffer are shown in black and orange, respectively, and are largely superimposable. Blue and red traces represent the interaction of NM and HM duplexes, respectively, with SRA (7.5 μM). The solid grey lines show the fits of the data to equation (3). The insets in panels A–D display the zoomed early-time region in order to highlight the fast kinetic phases observed with the ^th^G-labelled NM duplexes and the HM duplex labeled at position 6′. Panels E–H show the observed rate constants *k*_obs1_ (♦) and *k*_obs2_ (▪) as a function of the [SRA]/[DNA] concentration ratio for HM (red) and NM (blue) duplexes. The solid lines representing the average values, indicate that the *k*_obs_ values are independent of SRA concentration. Enlarged versions of the insets are provided in Supplementary Fig. S10A–D, together with a zoom of the kinetic traces of the free duplexes.

We next investigated the kinetics of the interaction of SRA with ^th^G-labelled HM duplexes (Fig. 4A–D, red traces) and NM duplexes (Fig. 4A–D, blue traces) using the fluorescence stopped-flow technique. It should be kept in mind that the time scales for absorption (attoseconds) and fluorescence (nanoseconds) being several orders of magnitude faster than the millisecond recording time of our stopped-flow instrument, the formation of the various complexes is associated with “simultaneous and instantaneous” changes in absorption and emission signals. Comparison with the traces recorded in the absence of protein (Fig. 4A–D, black curves) revealed an initial fluorescence increase too fast to be resolved, suggesting the presence of a kinetically unresolvable step. This unresolvable step was clearly evidenced for the interaction of both ^th^G-labelled HM and NM duplexes with SRA G448D, which binds duplexes but lacks base flipping activity [18] (Supplementary Fig. S8). With this mutant, ^th^G fluorescence changes at all four positions occurred exclusively within the dead time of the stopped-flow setup, resulting in a flat time trace (Supplementary Fig. S9A–D, red and blue traces). Furthermore, the close similarity of the kinetic traces in each panel of Supplementary Fig. S9 confirmed that SRA G448D interacts in the same way with NM and HM duplexes. Notably, kinetically unresolvable steps are frequently observed in protein–DNA interactions and are attributed to a very rapid non-specific encounter followed by protein sliding and binding to its target site [26, 74–77].

It should be kept in mind that the changes in the kinetic traces of SRA interaction with NM duplexes (insets of Fig. 4A–D and Supplementary Fig. S10A–D) revealed a sharp fluorescence increase, governed by an observed rate constant, *k*_obs1_ (300–600 s^–1^), independent of SRA concentration and the labelled position. This suggests that all ^th^G positions monitor the same first order molecular event. Moreover, by including the initial non-resolvable step as an adjustable fluorescence intensity *I*_i_ at *t* = 0 in equation 3, we found that the ratio of *I*_i_ to *I*_0_, the intensity of the free duplex, was well consistent with the fluorescence change observed upon binding of SRA G448D to the labelled duplexes (Fig. 3D). This confirms an unresolvable step for SRA, which probably describes the same initial binding process as SRA G448D.

For SRA interaction with HM duplexes, the kinetic traces were clearly biphasic (Fig. 4A, B, and D) with the exception for ^th^G at 6′ position, where the kinetic trace is parallel to the trace obtained with the corresponding NM duplex (Fig. 4C). The initial unresolvable step as well as the *k*_obs1_ values (Fig. 4E–H) and associated fluorescence changes (Supplementary Fig. S11) of the fast resolvable phase were similar to those with NM duplexes, suggesting that they describe the same molecular events. The *k*_obs2_ values governing the slow phase only observed with HM duplexes were found to be independent of SRA concentration, but not of ^th^G position with the following order: ^th^G7 (14 ± 1 s^–1^) < ^th^G8’ (24 ± 1 s^–1^) < ^th^G5 (44 ± 2 s^–1^). In order to exclude the possibility that ^th^G at different positions perturbs the interaction to different extents, we monitored the kinetic traces of SRA interaction with NM and HM duplexes doubly labelled at positions 7 and 8′ (Supplementary Fig. S12). While the *k*_obs1_ value (540 s^–1^) closely matches the *k*_obs1_ values of all singly labelled HM and NM duplexes, the *k*_obs2_ value (16.8 s^-1^) of the HM duplex appears as an average of the values observed for ^th^G at position 7 and 8′, which are too close to be resolved. This observation excludes that ^th^G significantly perturbs the two kinetic phases and confirms that at least one kinetic step is slower at position 7 as compared to position 8′. In contrast to the rather constant (2-fold) increase in fluorescence induced by the fast resolvable step at all four ^th^G positions, the fluorescence change associated with the slow phase varies with ^th^G position, being 2.3-, 2-, and 1.5-fold at positions 7, 8′, and 5, respectively. The absence of a slow step with HM duplexes labelled with ^th^G6′, while the binding specificity of SRA to HM duplexes is preserved (Fig. 2A), suggests that the fluorescence signal associated with ^th^G at this position is insensitive to the slow step.

**Figure 5.**
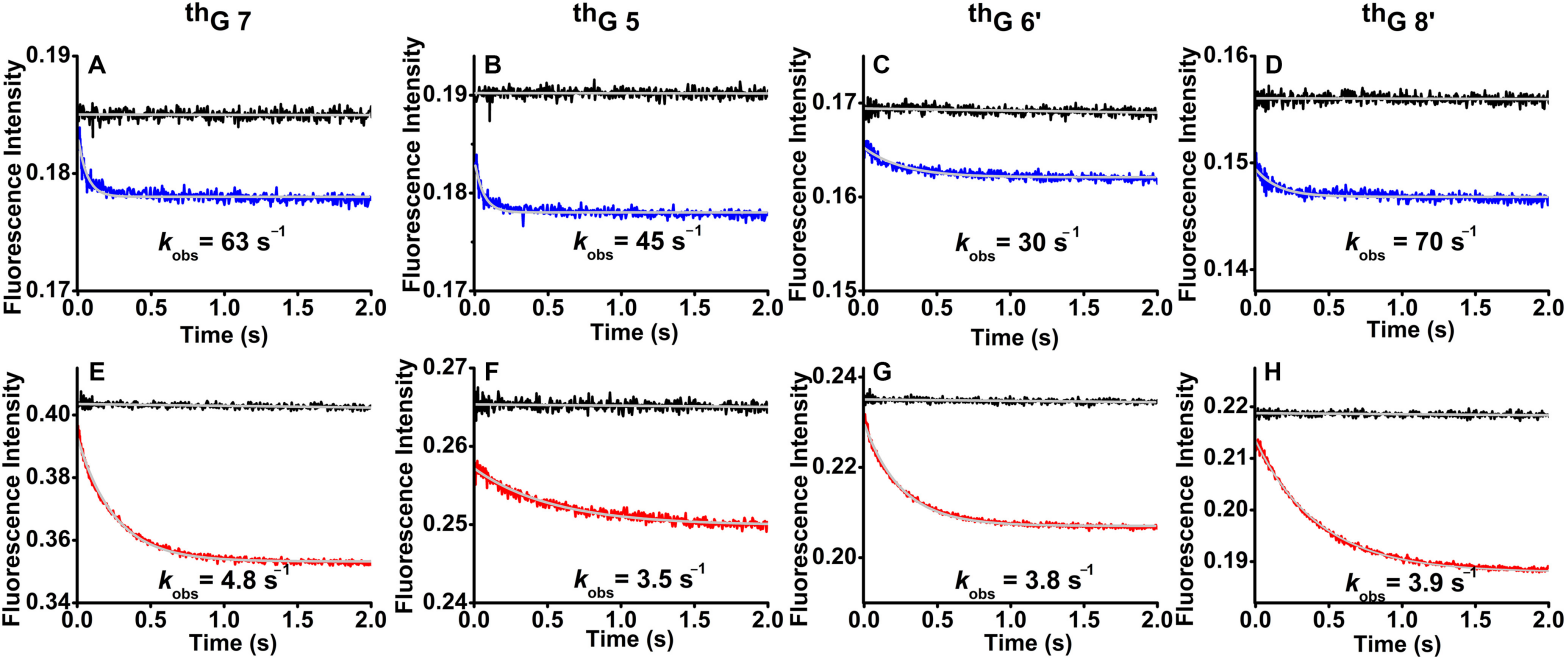
Dissociation kinetics of the complexes of SRA bound to HM and NM duplexes, as monitored by stopped-flow fluorescence measurements. Dissociation was triggered by mixing the pre-formed protein–DNA complexes with a large excess of calf thymus DNA. (**A**–**D**) Dissociation kinetic traces for NM duplexes. Each panel shows the dissociation trace of the complexes of SRA with ^th^G-labelled NM duplexes upon addition of calf thymus DNA (blue trace) or buffer (black trace). Complexes were prepared using 0.6 μM NM duplex and 3.0 μM SRA prior to mixing with an equal volume of 1500 μM calf thymus DNA. The grey lines are the single-exponential fits to the dissociation traces using the observed rate constant (*k*_obs_) given in the panels. (**E**–**H**) Dissociation kinetic traces for HM duplexes. Complexes were prepared with 0.6 μM HM duplex and 1.8 μM SRA prior to mixing with an equal volume of 1500 μM calf thymus DNA. The black traces show SRA/HM duplex complexes mixed with buffer, while the red traces show their dissociation upon addition of calf thymus DNA. The grey lines are the single-exponential fits to the dissociation traces using the observed rate constant (*k*_obs_) given in the panels. All experiments were performed in 20 mM PBS (pH 7.5), 50 mM NaCl, 2.5 mM TCEP, and 0.05% PEG20000, at 20 °C.

Dissociation experiments were next performed by adding a large excess of calf thymus DNA to preformed complexes of SRA with ^th^G-labelled HM or NM duplexes (Fig. 5). For both NM and HM duplexes, the fluorescence decay traces were adequately fitted to a single-exponential function. The values of the observed dissociation rate constants were found to marginally depend on the position of ^th^G and to be one order of magnitude higher for NM duplexes (30–70 s^–1^) as compared to HM duplexes (3–5 s^–1^). These rate constants could be considered as the rate limiting steps for the dissociation of the two complexes and therefore, be used to constraint the kinetic model.

### Modelling the kinetics of SRA interaction with NM and HM duplexes

**Scheme 1.**
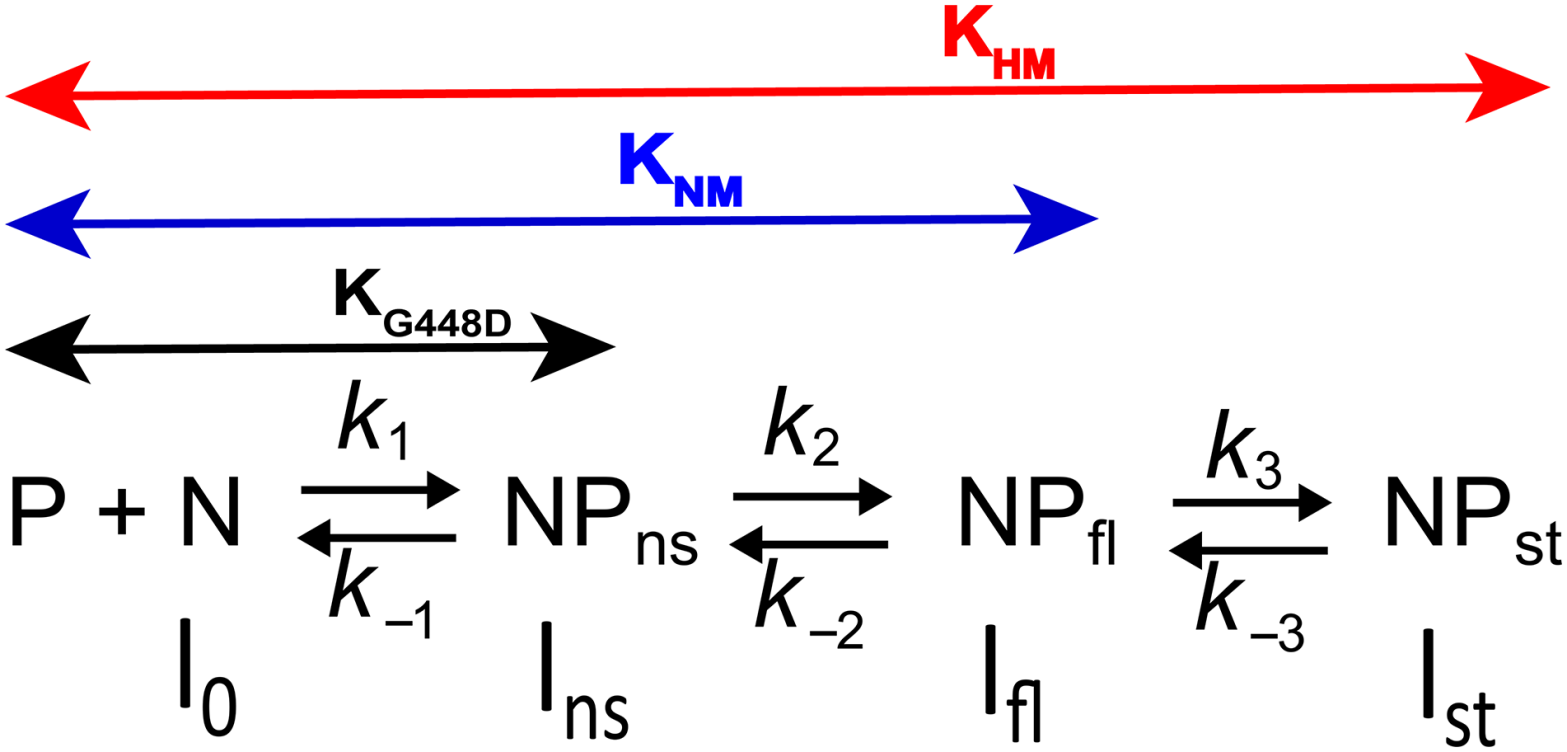
Kinetic model describing the interaction of SRA with HM and NM duplexes. P and N describe the free protein and ^th^G-labeled duplex, respectively. NP_ns_ describes the complex after the initial non-specific binding. NP_fl_ corresponds to the final complex with NM duplexes, but an intermediate complex with HM duplexes. NP_st_ corresponds to the final stable complex with HM duplexes. The intensities of N, NP_ns_, NP_fl_, and NP_st_ are denoted *I_x_*, where *x* = 0, ns, fl, and st, respectively .

Based on the data presented above, we established reaction Scheme 1, where the bimolecular step with rate constants *k*_1_ and *k*_–1_ is assumed to represent non-specific binding to the duplex (N) and subsequent sliding of the protein (P) towards the CpG site, resulting in the formation of the NP_ns_ intermediate. This first step is common to SRA and SRA G448D interactions with both NM and HM duplexes. As this step is the only one observed for SRA G448D, it follows that *k*_+1_/*k*_–1_ = *K*_G448D_, the association constant shown in Fig. 2B, while *I*_ns_, the fluorescence intensity of the non-specific NP_ns_ complex, is deduced from Fig. 3D. The second step governed by the forward *k*_2_ and backward *k*_–2_ rate constants is common for SRA interaction with NM and HM duplexes, leading to the NP_fl_ species, which is the final species for NM duplexes, but an intermediate for HM duplexes. Therefore, it can be deduced that *K*_G448D_$ \cdot$*k*_2_/*k*_–2_ = *K*_NM_, the association constant of SRA to NM duplexes (Fig. 2A, blue bars) and *I*_fl_, the fluorescence intensity of the resulting NP_fl_ complex, is deduced from Fig. 3B (blue bars). Finally, in HM duplexes, the third step with rate constants *k*_3_ and *k*_–3_ leads to the final stable complex NP_st_, whose structure has been solved [18]. Since the formation of NP_st_ includes the two first steps observed with NM duplexes, it immediately follows that *K*_NM_$ \cdot$*k*_3_/*k*_–3_ = *K*_HM_, the association constant of SRA to HM duplexes (Fig. 2A, red bars) and *I*_st_, the fluorescence intensity of NP_st_ is deduced from Fig. 3B (red bars).

Since *k_obs_*_1_ is independent of SRA concentration, the kinetically resolved steps 2 and 3 (Scheme 1) can be separated from the initial binding step, which is obviously concentration-dependent. Accordingly, we can assume that the first binding step is fully equilibrated within the dead time of the instrument and not reflected in the subsequent steps. In addition, since step 2 is much faster than step 3 (*k*_obs1_ >> *k*_obs2_), the *k*_obs1_ and *k*_obs2_ values are related to the rate constants of steps 2 and 3, according to [78]:


(4) \begin{eqnarray*}
{k_{{\mathrm{obs}}1}} = {k_2} + {k_{ - 2}}\hbox{ and }{k_{{\mathrm{obs}}2}} = \frac{{{k_3}\frac{{{k_2}}}{{{k_{ - 2}}}}}}{{1 + \frac{{{k_2}}}{{{k_{ - 2}}}}}} + {k_{ - 3}}
\end{eqnarray*}

**Figure 6.**
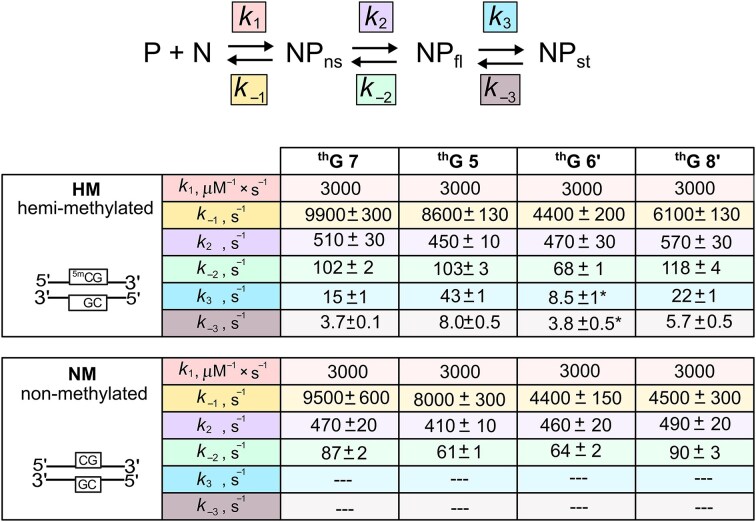
Kinetic rate constants for the interaction of SRA with HM and NM duplexes labelled with ^th^G at four different positions. The kinetic rate constants were obtained from the global fit of the kinetic traces in Supplementary Fig. S13 using the Dynafit software and the kinetic model shown above. As mentioned in the text, the *k*_1_ value was set to 3 × 10^9^ M^–1^s^–1^, but the values of all other rate constants were let floated. To enhance visualization, different colours are used to represent the various rate constants. *The *k*_3_ value for the ^th^G 6′ HM duplex could not be directly determined due to the absence of a detectable slow kinetic phase. Instead, *k*_3_ was calculated by: *k*_3_ = *K*_a_*k*_–1_*k*_–2_*k*_–3_/(*k*_1_*k*_2_) where *K*_a_ is the equilibrium association constant of SRA for the ^th^G6′ HM duplex given in Fig. 2A and the *k*_-3_ value was taken from Fig. 5G.

The initial rapid binding step must be included in the analysis, as it controls the amount of NP_ns_ intermediate entering the unimolecular steps. As Dynafit does not support the analysis of kinetic models in which a step is described by an equilibrium constant instead of its kinetic rate constants, we were forced to make assumptions about the rate constants *k*_1_ and *k*_–1_ associated with the initial step. Since this step governs the interaction of SRA G448D with the duplexes, the *k*_1_ value was arbitrarily set at 3 × 10^9^ M^–1^s^–1^, an intermediate value between the lower limit of 5 × 10^8^ M^–1^s^−1^, determined from the lowest SRA G448D concentration (2 μM) used in the experiment and the largest *k*_obs_ value (1000 s^–1^) measurable in our instrument, and the upper limit of 10^10^ M^–1^s^–1^ expected for diffusion-controlled DNA binding [26, 33]. Using equations (4) and the above-mentioned relationships between equilibrium association constants and kinetic rate constants to provide initial estimates for the rate constant values, we numerically fitted all kinetic traces of SRA interaction with HM and NM duplexes within the frame of Scheme 1 using Dynafit software (Supplementary Fig. S13). As can be seen from the residuals, excellent fits were obtained with both NM and HM duplexes for all the labelled positions, with fitted rate constants (Fig. 6) that deviate marginally from the initial estimates. This excellent fit in an overdetermined system with a constrained set of kinetic rate constants gives great confidence in the model used and the values of the rate constants.

Importantly, *k*_2_ values are close to the values of the rate constants governing the base flipping activity of many enzymes [25–28, 30, 31, 35, 38], strongly suggesting that this step could be attributed to the base flipping of the C/5mC residue at position 6 in NM/HM duplexes. Determination of *k*_2_ values from the fitting of the kinetic traces is a major step forward, in comparison to our preliminary study [45], where this step could not be resolved due to the technical limitation of the previous generation stopped-flow set up. In contrast to the *k*_2_ values, the values of the *k*_3_ rate constant governing the slow phase depended on ^th^G position, being of 43 ± 1 s^–1^, 22 ± 1 s^–1^, 15 ± 1 s^–1^, and 8.5 ± 1 s^–1^, for ^th^G at positions 5, 8′, 7, and 6′ respectively. Given that similar slow and sometimes position-dependent kinetics have been described with base-flipping enzymes [28, 30, 32, 35, 38], the rate constant *k*_3_, by analogy, probably governs the progressive conformational changes occurring in the SRA/DNA complex after base-flipping.

### Molecular dynamics simulations and quantum mechanical calculations

To further investigate the SRA/duplex interaction and the associated changes in ^th^G emission, we performed MD simulations and QM calculations using the 3D structures available for the SRA/HM duplex [17–19]. Being highly demanding, QM calculations were focused on the duplex labelled by ^th^G at position 7, which exhibits the largest fluorescence changes upon interaction with SRA.

Up to three replicas of unbiased MD simulations were run for 500 ns in explicit water, starting from the available structure of SRA in complex with the HM duplex [18] as well as with the SRA-free HM duplex. The complexes between SRA and the HM duplex as well as the HM duplex labelled by ^th^G at position 7 were investigated by MD simulations, using the ^th^G parameters validated previously [52, 53]. MD results were analysed by multiple descriptors, including the root-mean square deviation (RMSD) along MD time and the calculation of distances between target residues and/or atoms. Finally, a representative frame was extracted from MD trajectories as the centroid of the most populated cluster of MD frames, by clustering MD trajectories with a hierarchic agglomerative algorithm. MD representative frames were used for structural interpretation and as input in QM calculations. Overall, we confirmed by MD that replacing G by ^th^G at position 7 has a marginal impact on the structure of the duplexes [52]. Indeed, the representative frames of SRA/HM complexes bearing G or ^th^G in position 7 are highly superimposable (Cα RMSD = 0.58 Å) as are the all-atom RMSD plots calculated along MD trajectories (Supplementary Fig. S14A and B).

**Figure 7.**
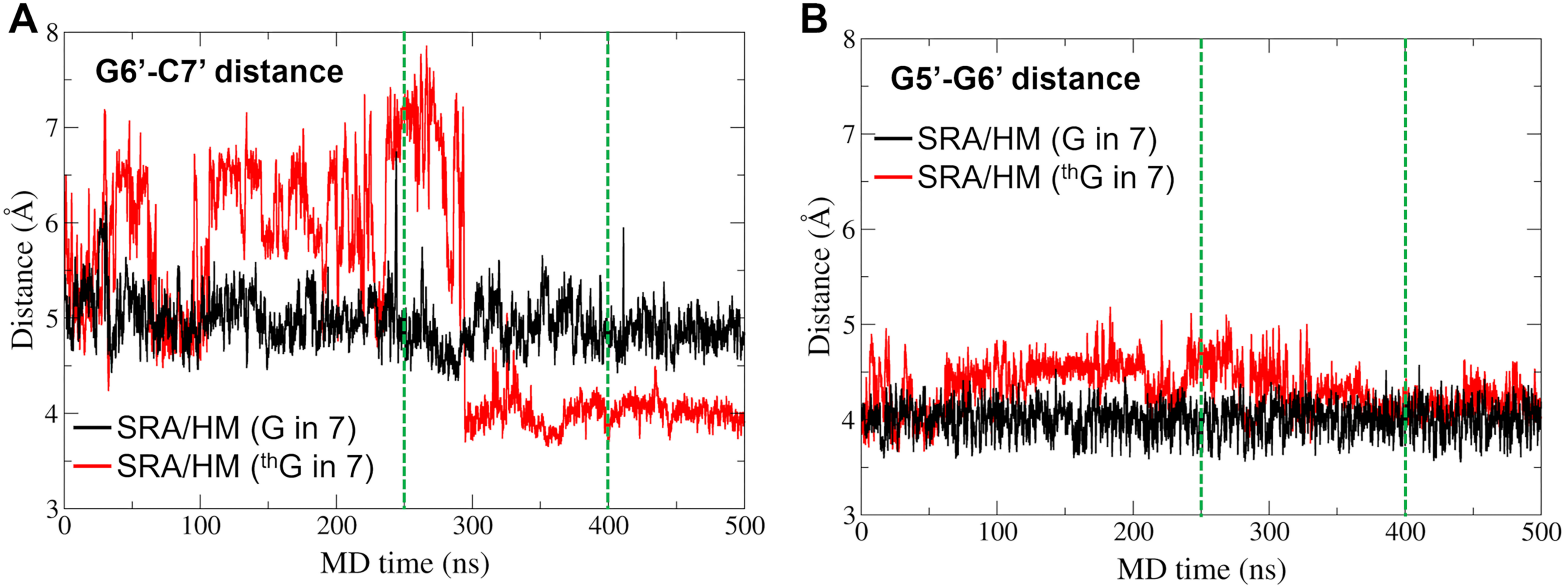
Distances between nucleobases G6′ and C7′ (**A**) and G5′ and G6′ (**B**) in the MD trajectories. The distances calculated in the SRA/HM complexes bearing G or ^th^G at position 7 are shown in black and red, respectively. The green dashed lines show the frames that were extracted for QM calculations, corresponding to a perturbed (250 ns) and a convergent (400 ns) conformation of the SRA/HM complex bearing ^th^G at position 7.

We then calculated inter-nucleotide distances along MD trajectories near position 7 to determine whether ^th^G could trigger local perturbations in the SRA/HM complex. Specifically, the G6′–C7′ distance is stable in the SRA/HM complex, but fluctuates when ^th^G replaces G7 (Fig. 7A). Following an increase in the distance within the first part of the MD trajectory, the distance decreases after ∼ 300 ns to reach a plateau value, slightly below the value observed in the SRA/HM complex bearing G7. As expected, this behaviour is also observed in the flanking pair (G5′–G6′) although in this case the deviation from the wild-type system is lower (Fig. 7B).

In summary, MD simulations suggest that ^th^G at position 7 has marginal impact on the overall structure and conformational stability of the SRA/HM complex, thus confirming its ability to properly mimic and replace G nucleotides. Nevertheless, ^th^G induces slight local perturbations, but *in silico* and biophysical studies indicated that these local changes marginally impact the properties and kinetics of the SRA/HM complex. Accordingly, two frames that describe the locally perturbed and the convergent conformation of the SRA/HM complex bearing ^th^G were extracted from MD trajectories at 250 and 400 ns, respectively (green dashed lines, Fig. 7). These two frames differ mainly by the structural behaviour of the G6′ base that is hydrogen bonded to the flipping 5mC. In the frame at 250 ns, G6′ is quite close to the opposite strand, and in particular to the sugar ring of ^th^G (distant only 3.4 Å). As a consequence, we label this frame as “G6′ close.” In contrast, in the frame at 400 ns, G6′ is farther from the opposite strand (distance from ^th^G sugar ring > 4.6 Å). This frame at 400 ns is labelled as “G6′ far.” These frames, together with the representative structures extracted by cluster analysis, were then submitted to QM calculations. To allow structural comparisons, a representative frame was also extracted from the MD trajectory of the SRA-free HM duplex, and was submitted to QM calculations as well.

QM calculations were performed to better understand the consequences of 5mC flipping on the spectroscopic properties of ^th^G. In particular, we wanted to rationalize the increase of the ^th^G fluorescence upon 5mC flipping and shed light on its dependence on the conformation of the two strands. On this basis, we could explain the complex dynamics revealed by stopped-flow data in term of the structural rearrangements of the duplex. We thus computed the absorption spectra of four representative DNA fragments (flipped or unflipped) and determined the emission properties of their excited state minima. A more extensive discussion on changes in the absorption spectra and related fluorescence data can be found in the Supplementary Information. As detailed below, in this case, the analysis of the computed absorption and emission spectra provide convergent indications, increasing the solidity of our interpretative framework.

**Figure 8.**
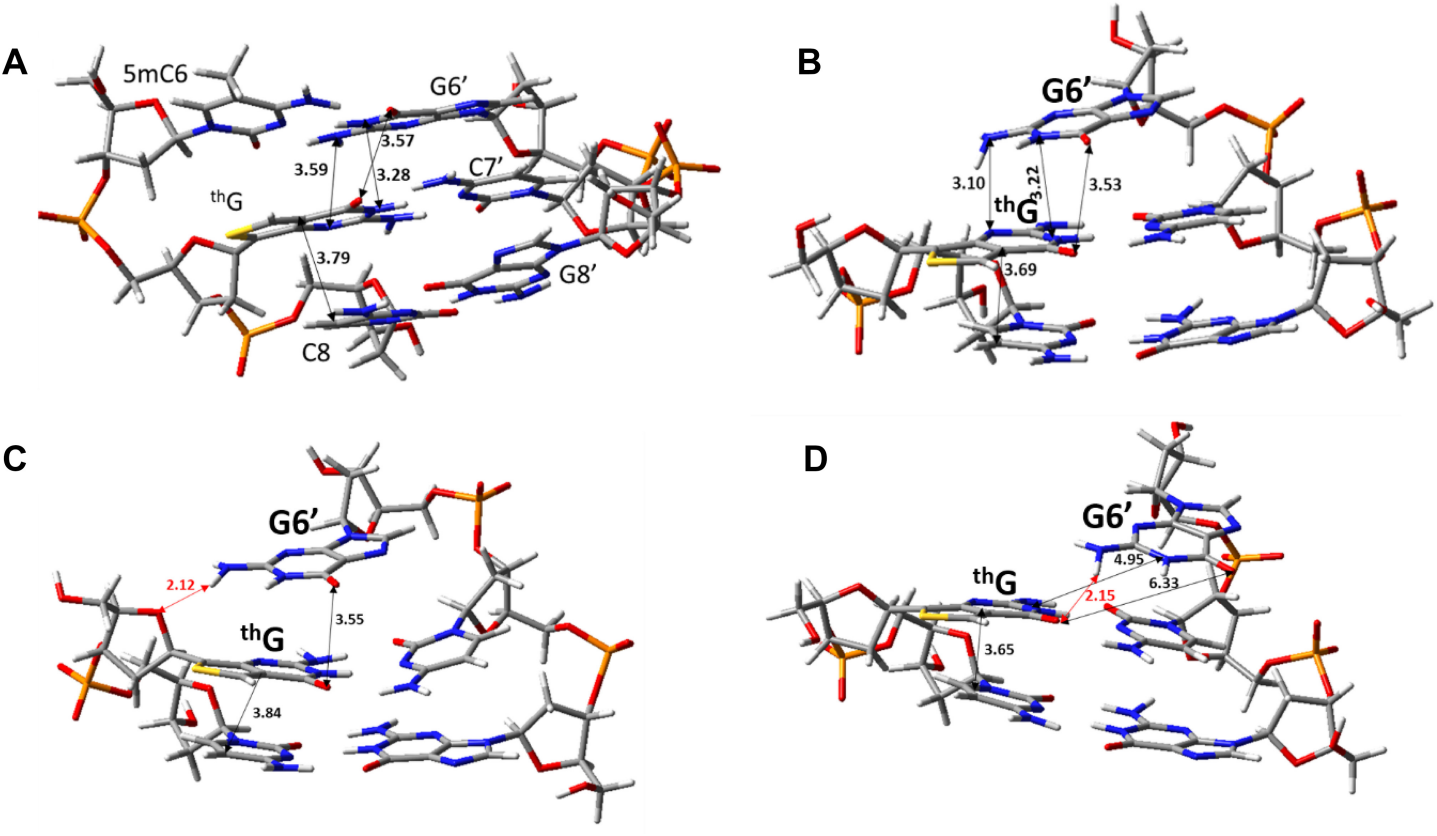
Optimized ground state electronic minima for (**A**) the 5mC^th^GC/GCG fragment from a representative structure of the MD simulation on the free duplex; (**B**) the ^th^GC/GCG fragment, starting from the non-flipped minimum and removing the 5mC base; the ^th^GC/GCG flipped fragment, starting from the frame at “G6′ close”-250 ns (**C**) or at G6′-far- 400 ns (**D**) of the MD simulation of the flipped duplex. Selected stacking distances (in Å) are reported.

As first step of our analysis, the 5mC^th^GC/GCG fragment (G at position 7 is replaced by ^th^G) was extracted from the representative structure of the SRA-free duplex obtained by MD simulation and then re-optimized (ground state minimum, Fig. 8A). This should be a representative structure of the non-flipped duplex, where ^th^G is stacked with 5mC. To simulate its absorption spectrum, we computed the transition energies of the lowest excited states for this fragment. As shown in Table 1 and Supplementary Table S1, S_1_ mainly corresponds to the lowest energy ππ* bright state localized on ^th^G (hereafter ^th^G*). S_2_ has a predominant character of a charge transfer (CT) state from ^th^G to C8, whereas S_3_ derives from the mixing between the bright ππ* excited state localized on 5mC (hereafter 5mC*) and the ^th^G→5mC CT state (Supplementary Fig. S15). S_4_ can be described as a CT state involving G8′ and C7′ (Supplementary Table S1), and S_5_ as ^th^G→5mC CT state.

**Table 1.** Adiabatic energies (VAE), in eV, oscillator strength (osc) and schematic description of the lowest energy excited states involving ^th^G, computed for the 5mC^th^GC or ^th^GC fragments of different representative structures^a^

	Non-flipped GS minimum		Flipped		Flipped (G6' close) frame 250 ns		Flipped (G6' far) frame 400 ns	
	VAE (osc)	Descr.	VAE (osc)	Descr.	VAE (osc)	Descr.	VAE (osc)	Descr.
S_1_	4.14 (0.15)	^th^G*	4.23 (0.19)	^th^G*	4.27 (0.19)	^th^G*	4.10 (0.18)	^th^G*
After opt	3.63 (0.17) **3.21**	^th^G*-min	3.73 (0.21) **3.28**	^th^G*-min	3.74 (0.21) **3.19** 3.62 (0.12) **3.10**	^th^G*-min ^th^G*/^th^GC-CT-min	3.58 (0.19) **3.13**	^th^G*-min
S_2_	4.74 (0.02)	^th^G→C8	4.99 (0.045)	^th^G→C8 + C8*	4.97 (0.00)	^th^G→C8	4.91 (0.05)	^th^G→C8
After opt	3.87 (0.01) **2.99**	^th^GC-CT-min	4.18 (0.02) **3.24**	^th^GC-CT-min		decay to ^th^G*-min		decay to ^th^G*-min
S_3_	5.11 (0.17)	5mC* + ^th^G→5mC						
S_5_	5.15 (0.01)	^th^G→5mC			5.14(0.02)^b^	G6’→^th^G		

^a^PCM/TD-M052X/6-31G(d) calculations in water. Emission energies are in bold. The non-flipped 5mCthGC/GCG fragment was extracted from the representative structure of the SRA-free duplex obtained by MD simulation and then re-optimized. The flipped state corresponds to the optimized thGC/GCG fragment of the SRA-free duplex, but where the 5mC residue has been removed. The flipped G6′ close and G6′ far structures correspond to optimized thGC/CG fragments obtained from two MD structures representative of two different arrangements of the flipped DNA in complex with SRA at 250 and 400 ns of the simulation time, respectively. See Supplementary Table S1 for additional data.

^b^This structure corresponds to S4.

Geometry optimization of S_1_ leads to a minimum localized on ^th^G* (^th^G*-min). S_2_ geometry optimization leads to ^th^G→C8 (^th^GC-CT-min), only 0.24 eV less stable than ^th^G*-min. In line with previous investigations [46, 52], the decrease in ^th^G fluorescence quantum yield can be associated to these non-emissive CT states involving stacked partners (5mC and C8), which can act as quenchers of the emission from ^th^G*-min.

To explore the photophysical features of ^th^G after 5mC base flipping and identify the possible determinants of the fluorescence changes associated with the slow step, we first optimized the ^th^GC/GCG fragment of the SRA-free duplex, but where the 5mC residue has been removed. As shown in Fig. 8B, the stacking geometry of G6′ is similar to that observed in Fig. 8A, although the amino group of G6′ is slightly closer to ^th^G. The lowest energy excited states computed in the minima (Table 1) are similar to those in the non-flipped duplex, but without excited states related to 5mC. Moreover, S_2_ has a smaller CT character, but with significant coupling to C8*. Similar to the non-flipped duplex, S_1_ geometry optimization leads to a minimum localized on ^th^G* (^th^G*-min). We also located ^th^GC-CT-min, which is 0.45 eV less stable than ^th^G*-min, i.e. its relative stability is lower than that of the 5mC-containing duplex. These results account for the increase of ^th^G* fluorescence. After flipping, the intrastrand CT states are less effective quenchers of ^th^G* fluorescence, as 5mC is absent and ^th^GC-CT-min is relatively less stable.

In the final step of our analysis, we explored the dependence of the spectral properties of ^th^G on the structural features of the opposite strand and optimized the ^th^GC/CG fragment starting from two MD structures representative of two quite different arrangements of the flipped DNA in complex with the protein (Fig. 7), i.e. at 250 (G6′ close) and 400 ns (G6′ far) of the simulation time. Geometry optimization of the former structure in the absence of the protein leads to the minimum shown in Fig. 8C, where G6′ is partially stacked on ^th^G, forming a hydrogen bond (in red in Fig. 8C) with the sugar of the opposite strand. In this structure, ^th^G and C8 exhibit a peculiar stacking geometry, with the pyrimidine ring of ^th^G almost in a perfect face-to-face arrangement. The lowest energy excited states of this minimum are described in Table 1 and Supplementary Table S1. While the three lowest energy excited states are similar to those found for the flipped duplex, we find a “new” excited state (S_4_) with a significant G6′→^th^G CT character in G6′ close (Supplementary Fig. S16). Excited state geometry optimizations provide a picture somewhat different from that for the unflipped structure. Indeed, S_1_ optimization converges to a minimum with significant CT character (as indicated by the smaller intensity), where the two pyrimidine rings of ^th^G and C8 get closer (Supplementary Figs S17A and S18). Likely due to the strong coupling with the ^th^G* excited state, it has not been possible to locate a minimum for the ^th^GC-CT, and decay to ^th^G* is always predicted (Supplementary Table S1). Interestingly, we also located a pure ^th^G* minimum (i.e. without a significant CT character), where G6′ keeps the hydrogen bond with the opposite strand, but ^th^G shows a smaller overlap with C8 (Supplementary Fig. S17B).

In the G6′ far frame at 400 ns, G6′ is poorly stacked with ^th^G (Fig. 8D) and, therefore, no excited states involving the two bases are found among the lowest energy ones (Table 1). Geometry optimization of S_1_ leads to ^th^G*-min, where the excitation is still well localized on ^th^G (Supplementary Fig. S19). Geometry optimization of S_2_ does not locate a real minimum for ^th^GC-CT and decay to ^th^G* is predicted.

In conclusion, by studying three different arrangements of a duplex lacking the 5mC base stacked with ^th^G, we obtain some clues about the structural rearrangements that follow the base flipping step and that are monitored by changes in ^th^G fluorescence associated with the slow kinetic step. Our calculations indicate that (i) in addition to stacking with 5mC, the photophysical properties of ^th^G are also sensitive to the conformation adopted by the G6′ residue opposite the “flipped” 5mC and that (ii) structural arrangements involving greater stacking between ^th^G and G6′ are possible, leading to an additional channel for fluorescence quenching, involving an excited state with strong G6′ → ^th^G CT character.

Finally, to investigate the origin of the small fluorescence increase accompanying the initial binding step, we compared the MD structure of the HM duplex complexed with SRA G448D (Supplementary Fig. S20) assumed to mimic NP_ns_ in Scheme 1 with the structure of the free duplex. This comparison revealed a significant displacement of the G6’ residue as a result of the insertion of the Arg491 residue of SRA G448D. This displacement increases the mean distance with ^th^G7 from 6.2 to 6.85 Å, which may in turn lead to a decrease in the CT-induced quenching processes and thus, explain the small fluorescence increase on NP_ns_ formation.

## Discussion

Base flipping is a key process used by many enzymes, such as DNA methyltransferases, DNA repair enzymes, and RNA modification enzymes, to chemically modify their substrates. UHRF1 does not alter DNA on its own, but uses the base flipping process induced by its SRA domain during HM DNA reading to recruit the methylation protein DNMT1 to mCpG sites [17, 19]. While the base flipping mechanism has been extensively studied in enzymatic reactions, information on its mechanism in non-enzymatic processes is scarce.

To investigate the base flipping mechanism of the SRA domain of UHRF1, we have used the FNA ^th^G that nearly perfectly substitutes G in DNA duplexes and sensitively reports on local conformational changes through parallel changes in absorbance and fluorescence linked to stacking-induced modulation of ^th^G’s electronic states [42, 44, 46, 52]. To obtain a full picture, we have substituted four positions within (7 and 6') or next to (5 and 8') the CpG site. Individually replacing the natural nucleobases with ^th^G in all four positions preserved the binding selectivity of SRA to HM duplexes compared to NM duplexes [71, 72, 79], confirming that ^th^G does not significantly alter the interaction with SRA (Fig. 2). While the base-flipping incompetent SRA G448D induced only limited change in the absorption and emission of ^th^G (20%–30%), two-fold changes in these features were observed for SRA binding to the ^th^G-labelled NM duplexes, and up to six-fold for binding to the ^th^G-labelled HM duplexes (Fig. 3).

**Figure 9.**
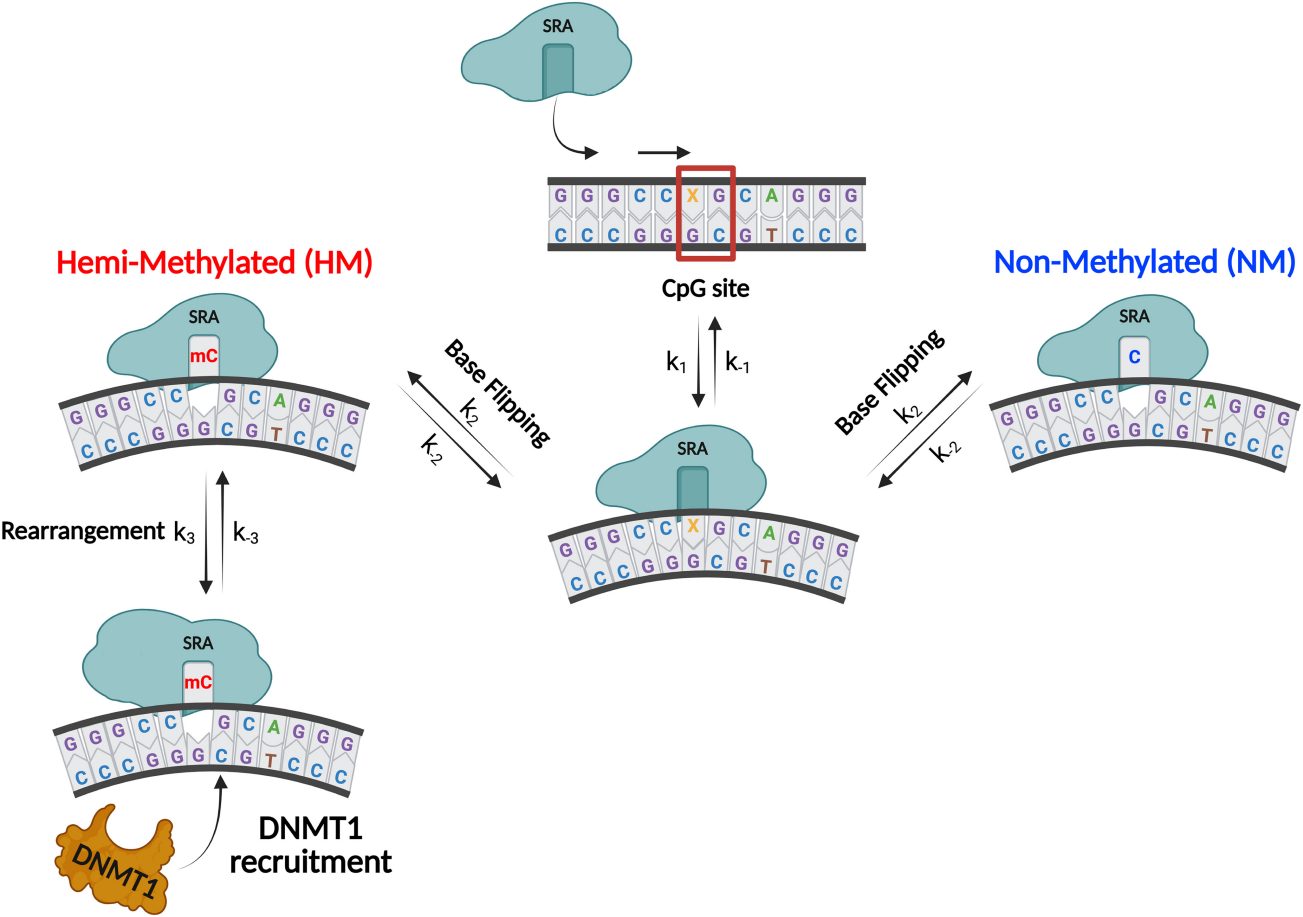
Proposed model illustrating the proof-reading function of the SRA domain of UHRF1 and its recruitment of DNMT1. The first step is assumed to correspond to non-specific binding and sliding of SRA to locate a CpG site on HM (X = 5mC) or NM (X = C) duplexes (central upper part). Upon finding a CpG site, SRA induces the flipping of 5mC or C into its dedicated binding pocket (central part). While C residues rapidly flip back, allowing the protein to continue the reading or detach (right part), HM duplexes with flipped 5mC undergo with bound SRA a slow rearrangement step that kinetically controls DNMT1 recruitment (left part).

Using stopped flow techniques, we deduced a two-step interaction model for NM duplexes and a three-step model for HM duplexes (Fig. 9). The first step, common to HM and NM duplexes, is tentatively attributed to SRA’s non-specific binding and further sliding towards the CpG site, as its high kinetic rate constant value (*k*_1_ = 3 × 10^9^ M^–1^s^–1^) is comparable to that observed for other base flipping proteins [25, 31, 35, 77, 80], where a similar process was reported. This value is too high for a collisional rate constant and can only be explained if the initial non-specific interaction of SRA with DNA is followed by facilitated diffusion [81–84]. This process consists primarily of two motions: (i) hopping, where the protein jumps off the DNA and undergoes 3D diffusion before associating to the same or a different segment of DNA and (ii) sliding where a protein diffuses along the major groove of DNA without losing contact. While hopping is more relevant for long DNA sequences, sliding is the preferred mode for short DNA sequences, such as the 12 bp-duplex used in our work. Sliding is a one-dimensional process, which considerably reduces the space to be covered and therefore speeds up the process of reaching a target site (the CpG site, in our case). In further support of the sliding hypothesis, based on the diffusion coefficients reported for well-known DNA-binding proteins [81], the estimated time to scan the maximum distance of six base pairs to reach the central four base pair binding site centred around the CpG motif in the 12 bp duplex [18] is <0.1 ms and therefore, well below the dead time of our stopped flow set-up. As suggested for several enzymes [85, 86] and by the 3D structure of the SRA complex with the HM duplex, the SRA peptide chain likely plays a major role in this first step by acting like a palm that locally bends and destabilizes the DNA backbone [18, 19], while the NKR finger and the Val 446 residue acting as a thumb contribute to the recognition of the CpG site. Further support for this sliding hypothesis will require additional experiments using for instance single molecule techniques with longer DNA sequences [81, 82].

This first step is followed by a unimolecular step common to HM and NM duplexes, associated with base flipping of the 5mC or C residue in the CpG site. The forward (*k*_2_ = 400–550 s^–1^) and backward (*k*_-2_ = 80–110 s^–1^) rate constants of this base flipping process are consistent with the corresponding values observed for UDG, M. EcoRI, Ecodam, and Endo VIII [25–28, 30–32, 35]. To our knowledge, this is the first report showing that CpGs with NM and methylated C residues are recognized and base flipped with similar rate constants. However, while C residues rapidly flip back, allowing the SRA to continue reading or dissociate, the complexes with flipped 5mC enter a slow step.

This last step, specific to HM duplexes, is governed by fairly slow forward (*k*_3_ = 14–35 s^–1^) and backward (*k*_–3_ = 3–18 s^–1^) rate constants and is attributed to a conformational rearrangement of the complex, previously observed for several base flipping enzymes. Unlike the base flipping rate constants, which are similar for a wide range of proteins, the kinetic rate constants for conformational rearrangements span a much larger range. While rearrangement rate constants similar to SRA were observed for the M.HaI T250G mutant [35], a faster forward rate constant (300 s^–1^) was observed for UDG [25, 26] and a much slower one (<1 s^–1^) for DNA MTase [30]. Moreover, our multiple labelling strategy suggested progressive SRA-induced conformational changes, being fastest at position 5 and slowest at position 6'. In the absence of a crystallographic structure for the complex of SRA with the NM duplex, a precise description of the conformational rearrangement is not possible. Nevertheless, a previous comparison of the structures of SRA complexed to HM duplex and free SRA revealed that the precisely organized 5mC pocket has a significantly smaller volume in the complex compared to that of the unbound SRA due to conformational changes of residues 464–473, 567–576, and 478–501 that include sidechain movement of the Tyr 466 and Tyr 478 side chains [18]. Some of these conformational changes likely participate in the slow rearrangement monitored by ^th^G fluorescence changes. Of note, the data obtained in this study are inconsistent with our previous two-step model, where a non-specific interaction step is followed by a slow base flipping step only observed with HM duplexes. Indeed, if SRA were unable to base flip the C residue in NM duplexes, it should, like SRA G448D, only interact non-specifically in a bimolecular reaction with the duplexes, which is inconsistent with (i) the independence of *k*_obs1_ on SRA concentration for all ^th^G positions (Fig. 4), (ii) the absence of resolvable kinetic phase with the SRA G448D mutant, and (iii) the differences in ^th^G-labelled NM duplex fluorescence changes induced by the two proteins. Moreover, the values of the kinetic rate constants associated with the base flipping step in the two-step model would be one order of magnitude lower than the corresponding values reported for a number of base flipping proteins [30–32, 35, 38]. Therefore, the three-step model is currently the simplest one that integrates and matches all our data, but more complex models including species not detected at low concentrations cannot be ruled out.

**Figure 10.**
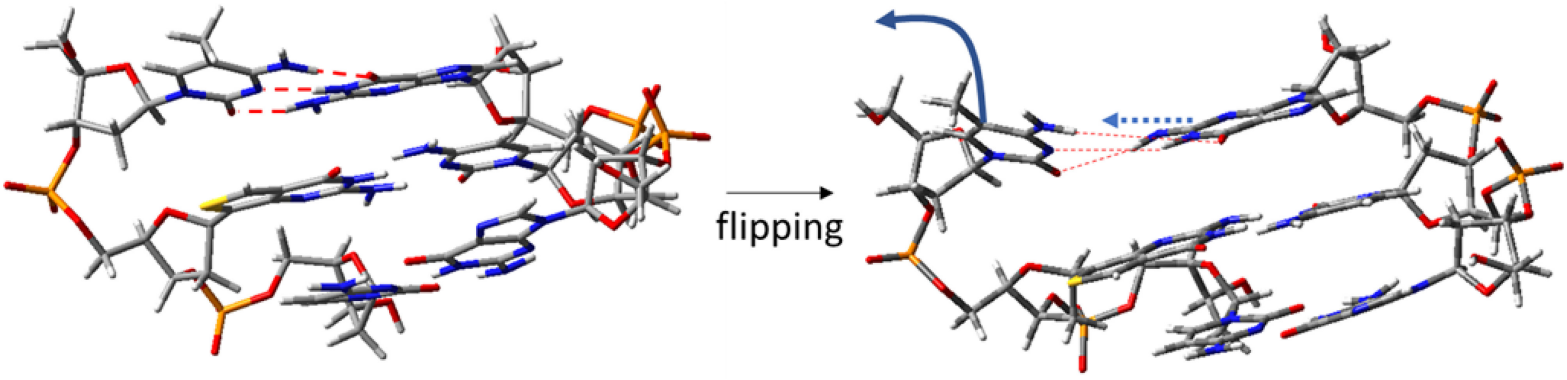
Possible model for the flipping intermediate (NP_fl_), obtained from QM calculations. Hydrogen bonds involving the flipping 5mC (whose motion is represented by the solid blue arrow) are depicted by dashed red lines. The motion of 5mC can induce a shift of its hydrogen bonded partner G, as suggested by the dashed blue arrow.

Based on our QM data, a possible model for the base-flipped intermediate NP_fl_ (Scheme 1) that leads to a two-fold increase in ^th^G fluorescence is suggested in Fig. 10. This intermediate is the result of C/5mC base flipping, which is assumed to be associated with a movement of G6', somewhat pulled by C/5mC, allowing hydrogen bonding interactions with 5mC to be maintained. This structure with G6'/^th^G stacking (Fig. 8C) would be characterized by a stronger fluorescence than the free duplex, where ^th^G is not solvent exposed and its fluorescence is quenched by several CT excited states (Table 1). The SRA/DNA complex then rearranges towards the 3D structure observed by X-ray crystallography, which is characterized by minimal G6'/^th^G stacking (Fig. 8D) and additional loss of CT excited states, explaining the further increase in ^th^G’s emission. Since G6′ interacts directly with Arg491, which acts both as a “mechanical wedge” to push 5mC or C out of the helix and a “stopper” to impede the back flipping process [26], this residue likely plays a major role in the rearrangement step. QM calculations indicate that the stacking geometry between ^th^G and C8 also matters. Arrangements characterized by a face-to-face stacking between the pyrimidine ring of ^th^G and the adjacent C8 base are indeed associated with quite a large electronic coupling between the ^th^G* and the ^th^G→C8 CT state, which could affect its fluorescence. It would therefore be possible that the NP_fl_ intermediate is characterized by such a stacking geometry (Fig. 8C), before reaching a structure with a smaller ring/ring overlap (Fig. 8D).

In this context, the question arises: why does 5mC promote conformational rearrangement, whereas C does not? It has been reported that in the binding pocket, the methyl group binding site forms a hemisphere with a radius of 2 Å that tightly fits the 5mC methyl group and can be unfavourably occupied by a water molecule when C replaces 5mC [18]. Since our data indicate comparable stability for the complex of SRA with NM duplex and the intermediate complex NP_fl_ with HM duplex, the fine adjustment of the methyl group in its binding site is probably a component of the rearrangement step. Another hypothesis raised previously to explain the preferential binding of SRA to HM duplexes is that the methyl group in 5mC reduces the dipole moment of cytosine, so that C is more stable than 5mC in free duplexes, while the opposite is true for SRA-bound duplexes [87]. This hypothesis is in variance with our data, which show that the final complex with the NM duplex and the intermediate complex with the HM duplex have the same binding constants. Further studies will be needed to clarify the role of 5mC in triggering the rearrangement step.

In conclusion, we have demonstrated the ability of SRA to flip C and 5mC residues indiscriminately. Importantly, only complexes with 5mC residues are further stabilized by a slower conformational rearrangement that leads to the crystallographically observed 3D structure [17–19]. Both the final structure of the complex and its rather long persistence time have been assumed to be crucial for recruiting DNMT1 to methylate the opposite C in the CpG site [45]. Our data suggest therefore that the base flipping process is used to proof read the DNA duplex in order to distinguish NM from methylated cytosines within the narrow SRA-binding pocket. Then, through a still elusive mechanism, the methyl group of 5mC within the binding pocket likely triggers [16, 41, 45, 72] the slow conformational rearrangement observed only for HM duplexes that controls DNMT1 recruitment and thus, the entire replication process of the methylation profile. Given that the HM and NM CpG sites differ by only a single methyl group, this conformational rearrangement step with limited impact on the thermodynamic binding constants appears to be an economical and effective way for the protein to control DNMT1 recruitment without compromising UHRF1 reading activity. Of course, this tentative mechanism will need to be confirmed in the context of the whole UHRF1 protein, in order to determine how the multiple domains of UHRF1 and its numerous interacting cell partners cooperate with the SRA domain to modulate the specificity for mCpG sites and impact the base flipping process.

## Supplementary Material

gkaf909_Supplemental_File

## Data Availability

The data supporting the conclusions of this study are provided in the main manuscript and the Supplementary Information file.

## Notes

Present address: Department of Chemistry, Indian Institute of Technology Hyderabad, Kandi, Sangareddy, Telangana 502284, India

## References

[1] Yan MS-C, Matouk CC, Marsden PA Epigenetics of the vascular endothelium. J Mol Biol. 2010; 109:215–24. 10.1152/japplphysiol.00131.2010. 20413423

[2] Xie S, Qian C The growing complexity of UHRF1-mediated maintenance DNA methylation. Genes. 2018; 9:600 10.3390/genes9120600. 30513966 PMC6316679

[3] Xue B, Zhao J, Feng P et al. Epigenetic mechanism and target therapy of UHRF1 protein complex in malignancies. OncoTargets Ther. 2019; 12:549–59. 10.2147/OTT.S192234. PMC6334784 30666134

[4] Cheng J, Yang Y, Fang J et al. Structural insight into coordinated recognition of trimethylated histone H3 lysine 9 (H3K9me3) by the plant homeodomain (PHD) and tandem tudor domain (TTD) of UHRF1 (ubiquitin-like, containing PHD and RING finger domains, 1) protein. J Biol Chem. 2013; 288:1329–39. 10.1074/jbc.M112.415398. 23161542 PMC3543016

[5] Hu L, Li Z, Wang P et al. Crystal structure of PHD domain of UHRF1 and insights into recognition of unmodified histone H3 arginine residue 2. Cell Res. 2011; 21:1374–8. 10.1038/cr.2011.124. 21808300 PMC3193472

[6] Baylin SB, Jones PA Epigenetic determinants of cancer. Cold Spring Harb Perspect Biol. 2016; 8:a019505 10.1101/cshperspect.a019505. 27194046 PMC5008069

[7] Jabbari K, Bernardi G Cytosine methylation and cpg, tpg (cpa) and tpa frequencies. Gene. 2004; 333:143–9. 10.1016/j.gene.2004.02.043. 15177689

[8] Feinberg AP, Ohlsson R, Henikoff S The epigenetic progenitor origin of human cancer. Nat Rev Genet. 2006; 7:21–33. 10.1038/nrg1748. 16369569

[9] Klose RJ, Bird AP Genomic DNA methylation: the mark and its mediators. Trends Biochem Sci. 2006; 31:89–97. 10.1016/j.tibs.2005.12.008. 16403636

[10] Sandoval J, Esteller M Cancer epigenomics: beyond genomics. Curr Opin Genet Dev. 2012; 22:50–5. 10.1016/j.gde.2012.02.008. 22402447

[11] Goldberg AD, Allis CD, Bernstein E Epigenetics: a landscape takes shape. Cell. 2007; 128:635–8. 10.1016/j.cell.2007.02.006. 17320500

[12] Suzuki MM, Bird A DNA methylation landscapes: provocative insights from epigenomics. Nat Rev Genet. 2008; 9:465–76. 10.1038/nrg2341. 18463664

[13] Murphy SK, Jirtle RL Imprinting evolution and the price of silence. Bioessays. 2003; 25:577–88. 10.1002/bies.10277. 12766947

[14] Okamoto I, Otte AP, Allis CD et al. Epigenetic dynamics of imprinted X inactivation during early mouse development. Science. 2004; 303:644–9. 10.1126/science.1092727. 14671313

[15] Bronner C, Alhosin M, Hamiche A et al. Coordinated dialogue between UHRF1 and DNMT1 to ensure faithful inheritance of methylated DNA patterns. Genes. 2019; 10:65 10.3390/genes10010065. 30669400 PMC6360023

[16] Bostick M, Kim JK, Estève P-O et al. UHRF1 plays a role in maintaining DNA methylation in mammalian cells. Science. 2007; 317:1760–4. 10.1126/science.1147939. 17673620

[17] Arita K, Ariyoshi M, Tochio H et al. Recognition of hemi-methylated DNA by the SRA protein UHRF1 by a base-flipping mechanism. Nature. 2008; 455:818–21. 10.1038/nature07249. 18772891

[18] Avvakumov GV, Walker JR, Xue S et al. Structural basis for recognition of hemi-methylated DNA by the SRA domain of human UHRF1. Nature. 2008; 455:822–5. 10.1038/nature07273. 18772889

[19] Hashimoto H, Horton JR, Zhang X et al. The SRA domain of UHRF1 flips 5-methylcytosine out of the DNA helix. Nature. 2008; 455:826–9. 10.1038/nature07280. 18772888 PMC2602803

[20] Adam S, Anteneh H, Hornisch M et al. DNA sequence-dependent activity and base flipping mechanisms of DNMT1 regulate genome-wide DNA methylation. Nat Commun. 2020; 11:3723 10.1038/s41467-020-17531-8. 32709850 PMC7381644

[21] Nishiyama A, Mulholland CB, Bultmann S et al. Two distinct modes of DNMT1 recruitment ensure stable maintenance DNA methylation. Nat Commun. 2020; 11:1222 10.1038/s41467-020-15006-4. 32144273 PMC7060239

[22] Sharif J, Muto M, Takebayashi S-i et al. The SRA protein Np95 mediates epigenetic inheritance by recruiting Dnmt1 to methylated DNA. Nature. 2007; 450:908–12. 10.1038/nature06397. 17994007

[23] Zhang J, Gao Q, Li P et al. S phase-dependent interaction with DNMT1 dictates the role of UHRF1 but not UHRF2 in DNA methylation maintenance. Cell Res. 2011; 21:1723–39. 10.1038/cr.2011.176. 22064703 PMC3357991

[24] Kuznetsov NA, Vorobjev YN, Krasnoperov LN et al. Thermodynamics of the multi-stage DNA lesion recognition and repair by formamidopyrimidine-DNA glycosylase using pyrrolocytosine fluorescence—stopped-flow pre-steady-state kinetics. Nucleic Acids Res. 2012; 40:7384–92. 10.1093/nar/gks423. 22584623 PMC3424566

[25] Jiang YL, Stivers JT Mutational analysis of the base-flipping mechanism of uracil DNA glycosylase. Biochemistry. 2002; 41:11236–47. 10.1021/bi026226r. 12220189

[26] Wong I, Lundquist AJ, Bernards AS et al. Presteady-state analysis of a single catalytic turnover by Escherichia coli uracil-DNA glycosylase reveals a “pinch-pull-push” mechanism. J Biol Chem. 2002; 277:19424–32. 10.1074/jbc.M201198200. 11907039

[27] Bellamy SR, Krusong K, Baldwin GS A rapid reaction analysis of uracil DNA glycosylase indicates an active mechanism of base flipping. Nucleic Acids Res. 2007; 35:1478–87. 10.1093/nar/gkm018. 17284454 PMC1865060

[28] Allan BW, Reich NO, Beechem JM Measurement of the absolute temporal coupling between DNA binding and base flipping. Biochemistry. 1999; 38:5308–14. 10.1021/bi9900020. 10220317

[29] Allan BW, Garcia R, Maegley K et al. DNA bending by EcoRI DNA methyltransferase accelerates base flipping but compromises specificity. J Biol Chem. 1999; 274:19269–75. 10.1074/jbc.274.27.19269. 10383435

[30] Liebert K, Hermann A, Schlickenrieder M et al. Stopped-flow and mutational analysis of base flipping by the *Escherichia coli* Dam DNA-(adenine-N6)-methyltransferase. J Mol Biol. 2004; 341:443–54. 10.1016/j.jmb.2004.05.033. 15276835

[31] Stivers JT, Pankiewicz KW, Watanabe KA Kinetic mechanism of damage site recognition and uracil flipping by *Escherichia coli* uracil DNA glycosylase. Biochemistry. 1999; 38:952–63. 10.1021/bi9818669. 9893991

[32] Allan BW, Beechem JM, Lindstrom WM et al. Direct real time observation of base flipping by the EcoRI DNA methyltransferase. J Biol Chem. 1998; 273:2368–73. 10.1074/jbc.273.4.2368. 9442083

[33] Wolfe AE, O’Brien PJ Kinetic mechanism for the flipping and excision of 1, N 6-ethenoadenine by human alkyladenine DNA glycosylase. Biochemistry. 2009; 48:11357–69. 10.1021/bi9015082. 19883114 PMC2792197

[34] Hendershot JM, O’Brien PJ Critical role of DNA intercalation in enzyme-catalyzed nucleotide flipping. Nucleic Acids Res. 2014; 42:12681–90. 10.1093/nar/gku919. 25324304 PMC4227769

[35] Vilkaitis G, Dong A, Weinhold E et al. Functional roles of the conserved threonine 250 in the target recognition domain of HhaI DNA methyltransferase. J Biol Chem. 2000; 275:38722–30. 10.1074/jbc.M005278200. 11102456

[36] Bernards AS, Miller JK, Bao KK et al. Flipping duplex DNA inside out: a double base-flipping reaction mechanism by *Escherichia coli* MutY adenine glycosylase. J Biol Chem. 2002; 277:20960–4. 10.1074/jbc.C200181200. 11964390

[37] Koval VV, Kuznetsov NA, Zharkov DO et al. Pre-steady-state kinetics shows differences in processing of various DNA lesions by Escherichia coli formamidopyrimidine-DNA glycosylase. Nucleic Acids Res. 2004; 32:926–35. 10.1093/nar/gkh237. 14769949 PMC373384

[38] Shieh F-K, Youngblood B, Reich NO The role of Arg165 towards base flipping, base stabilization and catalysis in M. HhaI. J Mol Biol. 2006; 362:516–27. 10.1016/j.jmb.2006.07.030. 16926025

[39] Kladova OA, Kuznetsova AA, Fedorova OS et al. Mutational and kinetic analysis of lesion recognition by *Escherichia coli* endonuclease VIII. Genes. 2017; 8:140 10.3390/genes8050140. 28505099 PMC5448014

[40] Pettersen EF, Goddard TD, Huang CC et al. UCSF Chimera—a visualization system for exploratory research and analysis. J Comput Chem. 2004; 25:1605–12. 10.1002/jcc.20084. 15264254

[41] Greiner VJ, Kovalenko L, Humbert N et al. Site-selective monitoring of the interaction of the SRA domain of UHRF1 with target DNA sequences labeled with 2-aminopurine. Biochemistry. 2015; 54:6012–20. 10.1021/acs.biochem.5b00419. 26368281

[42] Shin D, Sinkeldam RW, Tor Y Emissive RNA alphabet. J Am Chem Soc. 2011; 133:14912–5. 10.1021/ja206095a. 21866967 PMC3179766

[43] Park S, Otomo H, Zheng L et al. Highly emissive deoxyguanosine analogue capable of direct visualization of B–Z transition. Chem Commun. 2014; 50:1573–5. 10.1039/c3cc48297a. 24382561

[44] Sholokh M, Sharma R, Shin D et al. Conquering 2-aminopurine’s deficiencies: highly emissive isomorphic guanosine surrogate faithfully monitors guanosine conformation and dynamics in DNA. J Am Chem Soc. 2015; 137:3185–8. 10.1021/ja513107r. 25714036 PMC4357565

[45] Kilin V, Gavvala K, Barthes NP et al. Dynamics of methylated cytosine flipping by UHRF1. J Am Chem Soc. 2017; 139:2520–8. 10.1021/jacs.7b00154. 28112929 PMC5335914

[46] Ciaco S, Gavvala K, Greiner V et al. Thienoguanosine brightness in DNA duplexes is governed by the localization of its ππ* excitation in the lowest energy absorption band. Methods Appl Fluoresc. 2022; 10:035003 10.1088/2050-6120/ac6ab6. 35472854

[47] Grytsyk N, Richert L, Didier P et al. Thienoguanosine, a unique non-perturbing reporter for investigating rotational dynamics of DNA duplexes and their complexes with proteins. Int J Biol Macromol. 2022; 213:210–25. 10.1016/j.ijbiomac.2022.05.162. 35643159

[48] Krause M, Ukkonen K, Haataja T et al. A novel fed-batch based cultivation method provides high cell-density and improves yield of soluble recombinant proteins in shaken cultures. Microb Cell Factories. 2010; 9:11 10.1186/1475-2859-9-11. PMC2841585 20167131

[49] Lakowicz JR Principles of Fluorescence Spectroscopy. 2006; 3rd edn. New York Springer 10.1007/978-0-387-46312-4.

[50] Kuzmič P Program DYNAFIT for the analysis of enzyme kinetic data: application to HIV proteinase. Anal Biochem. 1996; 237:260–73. 8660575 10.1006/abio.1996.0238

[51] Salomon-Ferrer R, Case DA, Walker RC An overview of the Amber biomolecular simulation package. Wiley Interdiscip Rev Comput Mol Sci. 2013; 3:198–210. 10.1002/wcms.1121.

[52] Kuchlyan J, Martinez-Fernandez L, Mori M et al. What makes thienoguanosine an outstanding fluorescent DNA probe?. J Am Chem Soc. 2020; 142:16999–7014. 10.1021/jacs.0c06165. 32915558 PMC7544670

[53] Sholokh M, Improta R, Mori M et al. Tautomers of a fluorescent G surrogate and their distinct photophysics provide additional information channels. Angew Chem. 2016; 55:7974–8. 10.1002/anie.201601688. 27273741 PMC4978544

[54] Platella C, Ghirga F, Zizza P et al. Identification of effective anticancer G-quadruplex-targeting chemotypes through the exploration of a high diversity library of natural compounds. Pharmaceutics. 2021; 13:1611 10.3390/pharmaceutics13101611. 34683905 PMC8537501

[55] Ballone A, Picarazzi F, Prosser C et al. Experimental and computational druggability exploration of the 14-3-3ζ/SOS1pS1161 PPI interface. J Chem Inf Model. 2020; 60:6555–65. 10.1021/acs.jcim.0c00722. 33138374

[56] Case DA, Aktulga HM, Belfon K et al. AmberTools. J Chem Inf Model. 2023; 63:6183–91. 10.1021/acs.jcim.3c01153. 37805934 PMC10598796

[57] Hanwell MD, Curtis DE, Lonie DC et al. Avogadro: an advanced semantic chemical editor, visualization, and analysis platform. J Cheminform. 2012; 4:17 10.1186/1758-2946-4-17. 22889332 PMC3542060

[58] Špaĉková Na, Cheatham TE, Ryjáĉek F et al. Molecular dynamics simulations and thermodynamics analysis of DNA− drug complexes. Minor groove binding between 4′, 6-diamidino-2-phenylindole and DNA duplexes in solution. J Am Chem Soc. 2003; 125:1759–69. 12580601 10.1021/ja025660d

[59] Tian C, Kasavajhala K, Belfon KA et al. ff19SB: amino-acid-specific protein backbone parameters trained against quantum mechanics energy surfaces in solution. J Chem Theory Comput. 2019; 16:528–52. 10.1021/acs.jctc.9b00591. 31714766 PMC13071887

[60] Cheatham TE III, Case DA Twenty-five years of nucleic acid simulations. Biopolymers. 2013; 99:969–77. 10.1002/bip.22331. 23784813 PMC3820278

[61] Roe DR, Cheatham TE III PTRAJ and CPPTRAJ: software for processing and analysis of molecular dynamics trajectory data. J Chem Theory Comput. 2013; 9:3084–95. 10.1021/ct400341p. 26583988

[62] Schrodinger L The PyMOL molecular graphics system. Version. 2015; 1:8.

[63] Zhao Y, Schultz NE, Truhlar DG Design of density functionals by combining the method of constraint satisfaction with parametrization for thermochemistry, thermochemical kinetics, and noncovalent interactions. J Chem Theory Comput. 2006; 2:364–82. 10.1021/ct0502763. 26626525

[64] Zhao Y, Truhlar DG Density functionals with broad applicability in chemistry. Acc Chem Res. 2008; 41:157–67. 10.1021/ar700111a. 18186612

[65] Tomasi J, Mennucci B, Cammi R Quantum mechanical continuum solvation models. Chem Rev. 2005; 105:2999–3094. 10.1021/cr9904009. 16092826

[66] Miertuš S, Scrocco E, Tomasi J Electrostatic interaction of a solute with a continuum. A direct utilizaion of AB initio molecular potentials for the prevision of solvent effects. Chem Phys. 1981; 55:117–29. 10.1016/0301-0104(81)85090-2.

[67] Frisch MJ, Trucks GW, Schlegel HB et al. Gaussian 16 Rev.C.01. 2016; Wallingford, CT.

[68] Qian C, Li S, Jakoncic J et al. Structure and hemimethylated CpG binding of the SRA domain from human UHRF1. J Biol Chem. 2008; 283:34490–4. 10.1074/jbc.C800169200. 18945682 PMC2596396

[69] Rottach A, Frauer C, Pichler G et al. The multi-domain protein Np95 connects DNA methylation and histone modification. Nucleic Acids Res. 2010; 38:1796–804. 10.1093/nar/gkp1152. 20026581 PMC2847221

[70] Frauer C, Hoffmann T, Bultmann S et al. Recognition of 5-hydroxymethylcytosine by the Uhrf1 SRA domain. PLoS One. 2011; 6:e21306 10.1371/journal.pone.0021306. 21731699 PMC3120858

[71] Zhou T, Xiong J, Wang M et al. Structural basis for hydroxymethylcytosine recognition by the SRA domain of UHRF2. Mol Cell. 2014; 54:879–86. 10.1016/j.molcel.2014.04.003. 24813944

[72] Abhishek S, Nakarakanti NK, Deeksha W et al. Mechanistic insights into recognition of symmetric methylated cytosines in CpG and non-CpG DNA by UHRF1 SRA. Int J Biol Macromol. 2021; 170:514–22. 10.1016/j.ijbiomac.2020.12.149. 33359809

[73] Li X, Harris CJ, Zhong Z et al. Mechanistic insights into plant SUVH family H3K9 methyltransferases and their binding to context-biased non-CG DNA methylation. Proc Natl Acad Sci USA. 2018; 115:E8793–802. 30150382 10.1073/pnas.1809841115 PMC6140468

[74] Winter RB, Berg OG, Von Hippel PH Diffusion-driven mechanisms of protein translocation on nucleic acids. 3. The *Escherichia coli* lac repressor-operator interaction: kinetic measurements and conclusions. Biochemistry. 1981; 20:6961–77. 10.1021/bi00527a030. 7032584

[75] Slutsky M, Mirny LA Kinetics of protein–DNA interaction: facilitated target location in sequence-dependent potential. Biophys J. 2004; 87:4021–35. 10.1529/biophysj.104.050765. 15465864 PMC1304911

[76] Konttinen O, Carmody J, Kurnik M et al. High fidelity DNA strand-separation is the major specificity determinant in DNA methyltransferase CcrM’s catalytic mechanism. Nucleic Acids Res. 2023; 51:6883–98. 10.1093/nar/gkad443. 37326016 PMC10359602

[77] Hendershot JM, O’Brien PJ Transient kinetic methods for mechanistic characterization of dna binding and nucleotide flipping. Methods Enzymol. 2017; 592:377–415. 28668128 10.1016/bs.mie.2017.04.003

[78] Bagshaw CR Biomolecular Kinetics: A Step-by-step Guide. 2017; Boca Raton, FL, USA CRC Press 10.1201/9781315120355.

[79] Hashimoto H, Liu Y, Upadhyay AK et al. Recognition and potential mechanisms for replication and erasure of cytosine hydroxymethylation. Nucleic Acids Res. 2012; 40:4841–9. 10.1093/nar/gks155. 22362737 PMC3367191

[80] Hendershot JM, Wolfe AE, O’Brien PJ Substitution of active site tyrosines with tryptophan alters the free energy for nucleotide flipping by human alkyladenine DNA glycosylase. Biochemistry. 2011; 50:1864–74. 10.1021/bi101856a. 21244040 PMC3059348

[81] DeSantis MC, Li J-L, DeCenzo SH et al. Protein sliding and hopping kinetics on DNA. Biophys J. 2011; 100:192a 10.1016/j.bpj.2010.12.1268. PMC3683889 21405863

[82] Barsky D, Laurence TA, Venclovas Č How proteins slide on DNA. Biophysics of DNA-Protein Interactions: From Single Molecules to Biological Systems. 2011; New York, NY, USA Springer 39–68.

[83] DeSantis M, Li J, Wang Y Protein sliding and hopping kinetics on DNA. Phys Rev E. 2011; 83:021907 10.1103/PhysRevE.83.021907. PMC3683889 21405863

[84] Tafvizi A, Huang F, Fersht AR et al. A single-molecule characterization of p53 search on DNA. Proc Natl Acad Sci USA. 2011; 108:563–8. 10.1073/pnas.1016020107. 21178072 PMC3021058

[85] Stivers JT Site-specific DNA damage recognition by enzyme-induced base flipping. Prog Nucleic Acid Res Mol Biol. 2004; 77:37–65. 15196890 10.1016/S0079-6603(04)77002-6

[86] Klimasauskas S, Kumar S, Roberts RJ et al. Hhal methyltransferase flips its target base out of the DNA helix. Cell. 1994; 76:357–69. 10.1016/0092-8674(94)90342-5. 8293469

[87] Bianchi C, Zangi R How to distinguish methyl-cytosine from cytosine with high fidelity. J Mol Biol. 2012; 424:215–24. 10.1016/j.jmb.2012.09.024. 23041422

