# Supplementary material for: Decoding the base flipping mechanism of the SET- and RING-associated (SRA) domain of the epigenetic UHRF1 protein
Source: Nucleic Acids Res. 2025 Sep 17;53(17):gkaf909. doi: 10.1093/nar/gkaf909 (PMC12448856; doi:10.1093/nar/gkaf909)
Supplement: gkaf909_Supplemental_File [file gkaf909_supplemental_file.pdf]

Supporting Information

Decoding the base flipping mechanism of the SET- and RING-associated (SRA) domain of the epigenetic UHRF1 protein

Dipanjan Mukherjee[1], Stefano Ciaco[1, 2], Lara Martinez-Fernandez[3], Krishna Gavvala[1, §], Elisa Bombarda[1], Aurélie Bourdérioux[1], Dmytro Dziuba[1], Fabien Hanser[1], Nicolas Humbert[1], Aqib Javed[1], Marc Mousli[1], Pankhi Singh[1], Yitzhak Tor[4], Roberto Improta[5], Mattia Mori[2], Yves Mély*[1]

[1] Laboratoire de Bioimagerie et Pathologies, UMR 7021 CNRS Université de Strasbourg, Faculté de pharmacie, 74 route du Rhin, 67401 Illkirch, France

[2] Department of Biotechnology, Chemistry and Pharmacy, University of Siena, 53100 Siena, Italy.

[3] Departamento de Química, Facultad de Ciencias, Modúlo13, Universidad Autónoma de Madrid, Campus de Excelencia UAM-CSIC, Cantoblanco, 28049 Madrid, Spain.

[4] Department of Chemistry and Biochemistry, University of California, San Diego, La Jolla, CA, 92093-0358, USA.

[5] Consiglio Nazionale delle Ricerche, Istituto Biostrutture e Bioimmagini Via De Amicis 95, 80145, Napoli (Italy).

[§] Current address: Department of Chemistry, Indian Institute of Technology Hyderabad, Kandi, Sangareddy, Telangana-502284,India.

* Corresponding author: yves.mely@unistra.fr

Contents

**Figure S1**	**3**
**Figure S2**	**4**
**Figure S3**	**6**
**Figure S4**	**7**
**Figure S5**	**8**
**Figure S6**	**9**
**Figure S7**	**10**
**Figure S8**	**11**
**Figure S9**	**12**
**Figure S10**	**13**
**Figure S11**	**14**
**Figure S12**	**15**
**Figure S13**	**16-17**
**Figure S14**	**18**
**Additional discussion of the spectroscopic data and QM results**	**19- 21**
**Table S1**	**22**
**Figure S15**	**23**
**Figure S16**	**24**
**Figure S17**	**25**
**Figure S18**	**26**
**Figure S19**	**27**
**Figure S20**	**28**
**References**	**29**

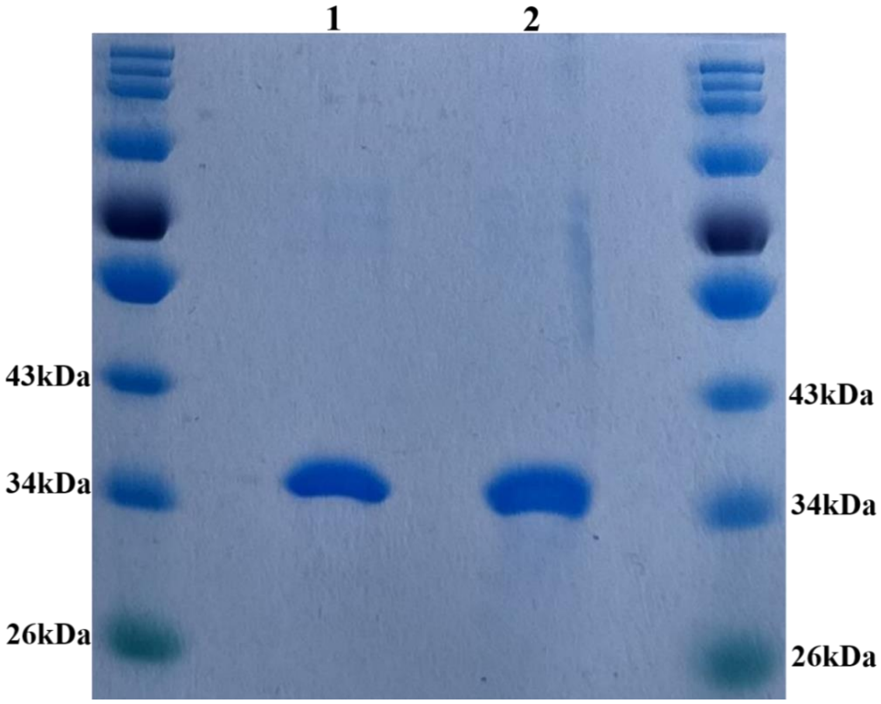

Figure S1: SDS-PAGE electrophoresis (10%) with Coomassie blue staining of about 3 μg of purified SRA (lane 1) and SRA G448D (lane 2) proteins. Both appear as a single broad band at about ~34 kDa. The leftmost and rightmost lanes show the Color Prestained Protein Standard, Broad Range (10–250 kDa) from New England Biolabs (catalog no. P7719S), used as a molecular weight marker.

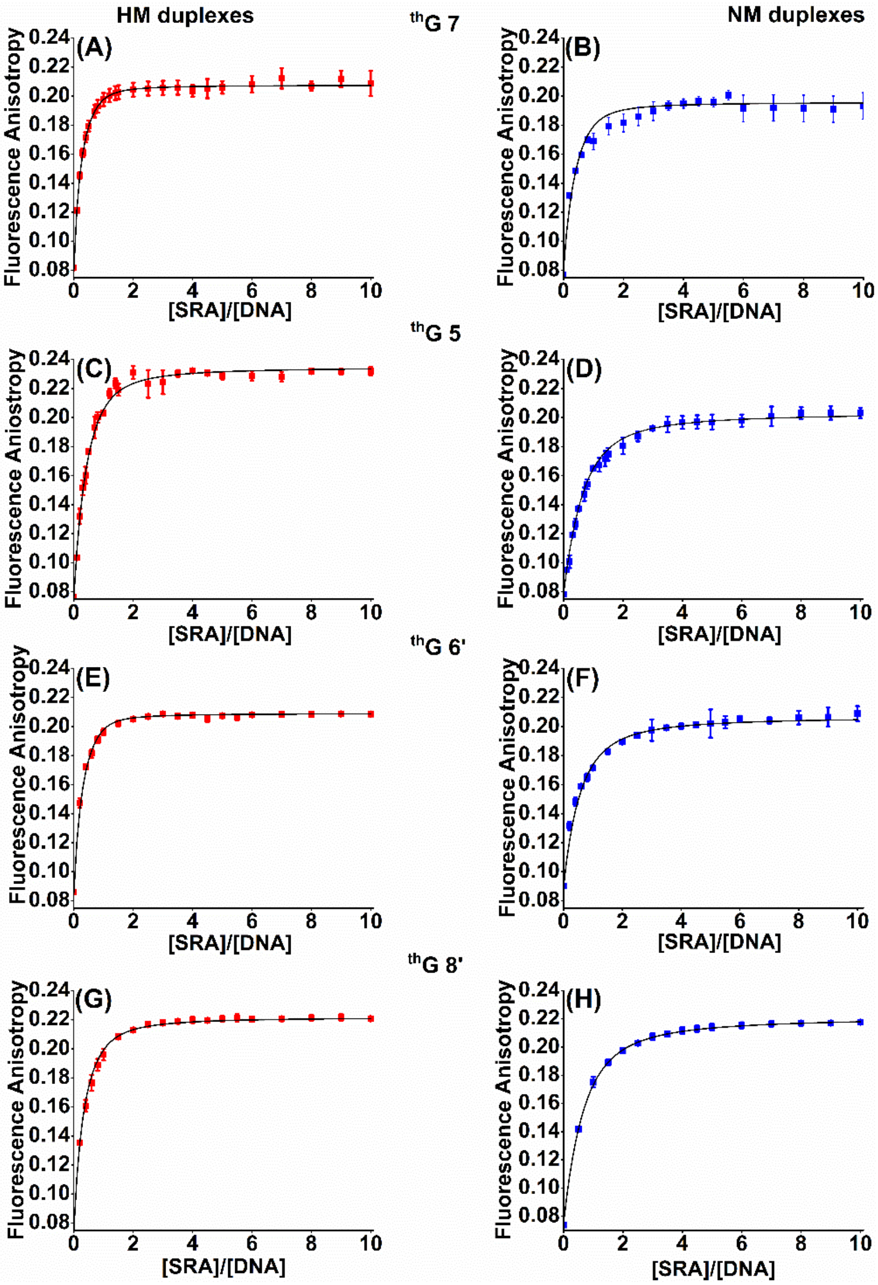

Figure S2: Titrations of thG-labelled HM (A, C, E and G) and NM (B, D, F and H) duplexes by SRA. The HM and NM duplexes were labelled by thG at position 7 (A, B), 5 (C, D), 6' (E, F) and 8' (G, H). The binding was monitored by fluorescence anisotropy (λ_{ex} = 350 nm and λ_{em} = 454 nm). The concentration of duplexes was 1 µM. Buffer was 20 mM PBS pH 7.5, 50 mM NaCl, 2.5 mM TCEP, 0.05% PEG 20000 at 20°C. Each data point (circle) and associated error bar are the mean and standard deviation for n=3 to 5 experiments. The solid lines are the fits of the experimental data to equations (1) and (2) with the binding constant values given in Figure 2A. For all positions of thG, the SRA affinity (2-3×10^6 M^{-1}) for NM duplexes was consistently 2–4-fold lower than for HM

duplexes. Of note, such binding preference was not observed for positions 6' and 8' in our previous work (main manuscript, reference 45), probably due to the non-optimized protein preparation protocol used at that time, which was leading to less active batches as compared to those obtained with the improved protocol used herein and described in the materials and methods section.

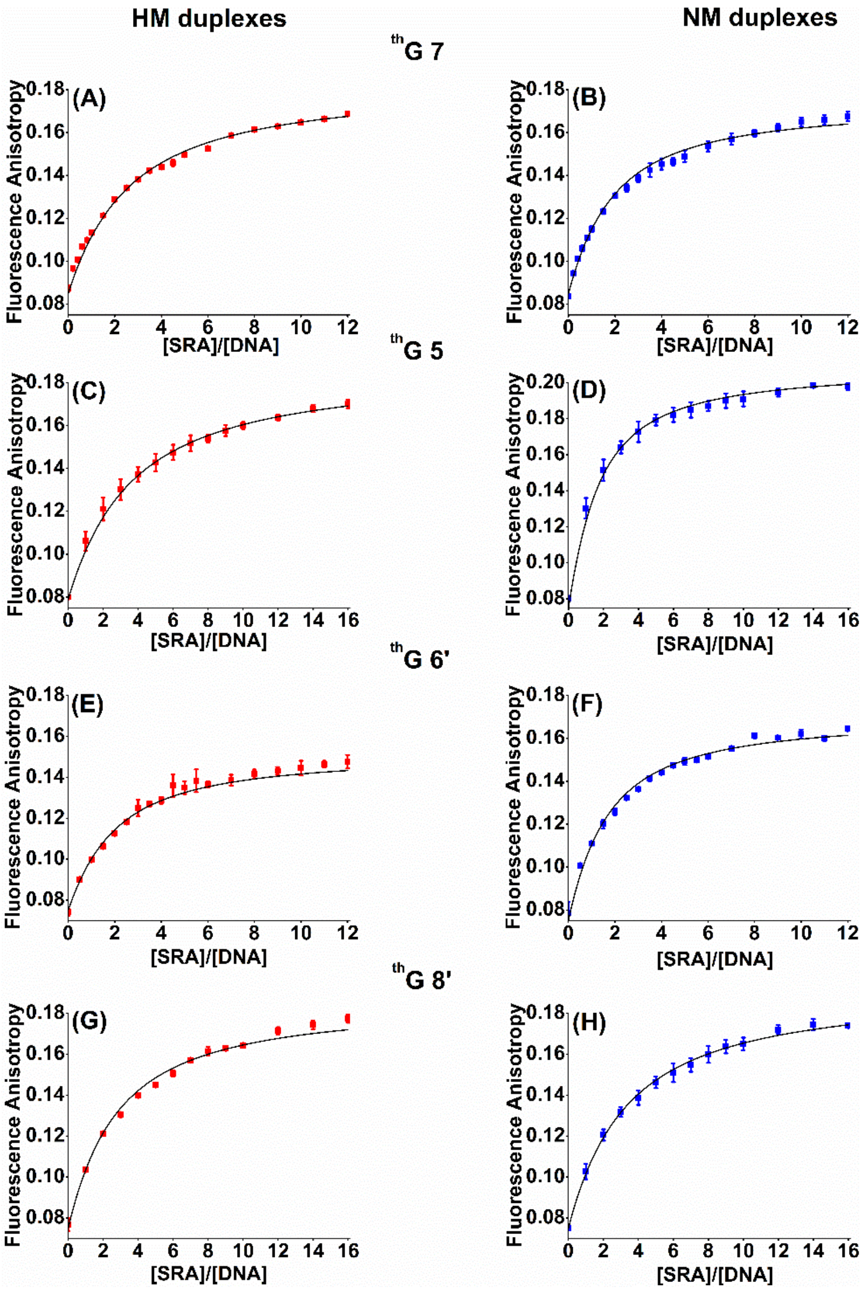

Figure S3: Titrations of thG-labelled HM (A, C, E and G) and NM (B, D, F and H) duplexes by SRA G448D. The HM and NM duplexes were labelled by thG at position 7 (A, B), 5 (C, D), 6' (E, F) and 8' (G, H). The binding was monitored by fluorescence anisotropy (λ_{ex} = 350 nm and λ_{em} = 454 nm). The concentration of duplexes was 1 µM. Buffer was as in Figure S2. Each data point (circle) and associated error bar are the mean and standard deviation for n=3 to 5 experiments. The solid lines are the fits of the experimental data to equations (1) and (2) using the binding constant values of Figure 2B.

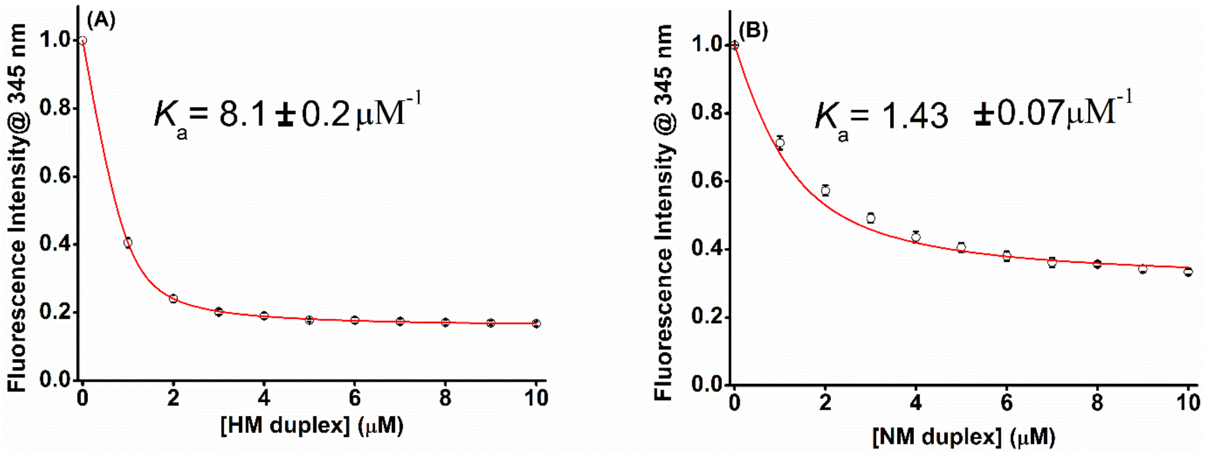

Figure S4: Titration of UHRF1-SRA by unlabelled HM (A) and NM (B) duplexes, as monitored using the intrinsic fluorescence of SRA tryptophan residues. Excitation was at 295 nm. Fluorescence intensity changes at 345 nm were recorded upon titration of SRA with HM and NM DNA duplexes. The concentration of SRA was fixed at 1 μM. All other experimental conditions were as in Figure S2. The fluorescence changes (squares) were fitted to the following equation using the K_a values given in the panels:

$$I_t = I_0 - ((I_0 - I_t)/L_t \times \frac{(1 + (L_t + n \times N_t) \times K_a) - \sqrt{(1 + (L_t + n \times N_t) \times K_a)^2 - 4 \times L_t \times n \times N_t \times K_a^2}}{2 * K_a}$$

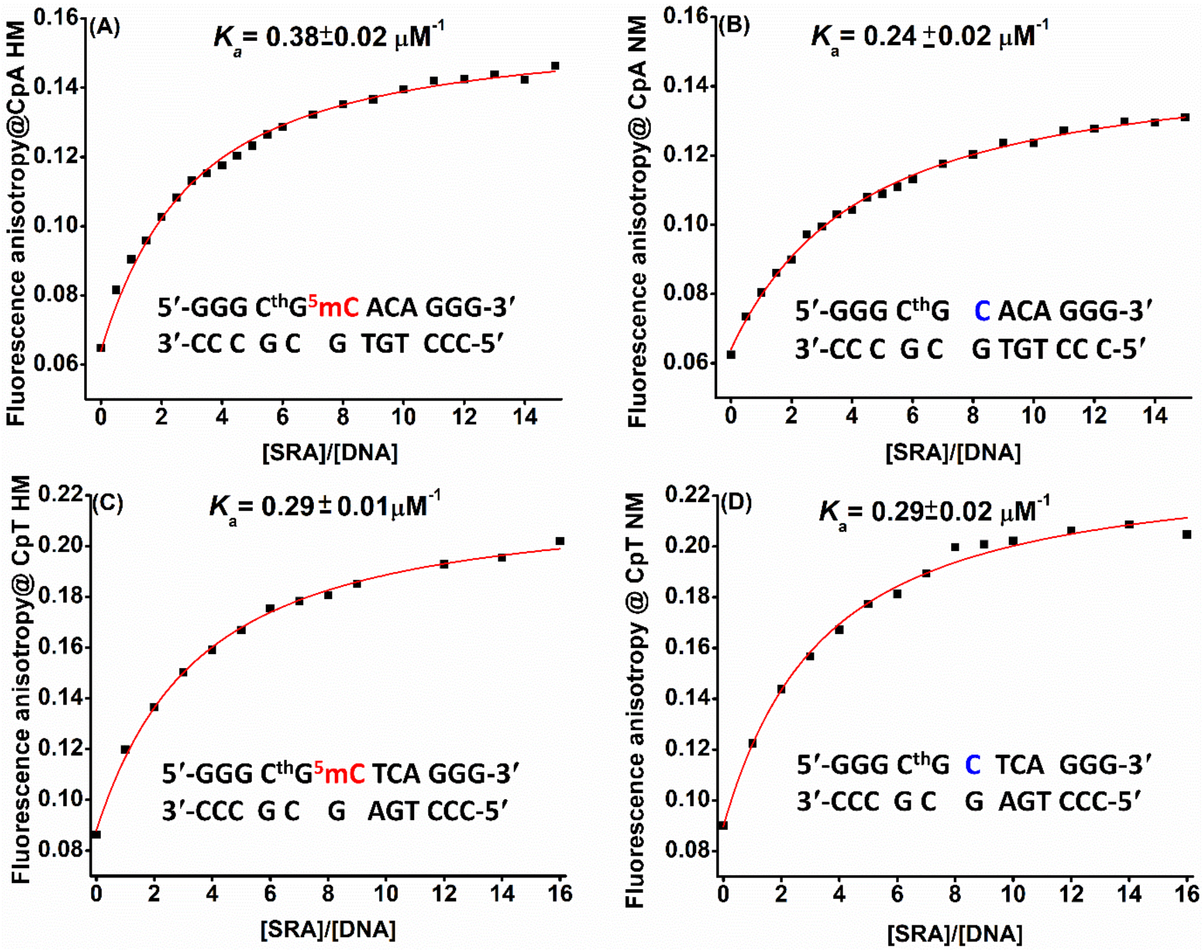

Figure S5: Titrations of thG-labelled HM and NM duplexes containing CpA and CpT motifs by SRA G448D. The binding was monitored by fluorescence anisotropy (λ_{ex} = 350 nm and λ_{em} = 454 nm). The concentration of duplexes was 1 μM. Buffer was as in Figure S2. The solid lines are the fits of the experimental data points (squares) to equations (1) and (2) using the indicated K_a values, given as mean +/- standard error of the mean for n=2 independent experiments.

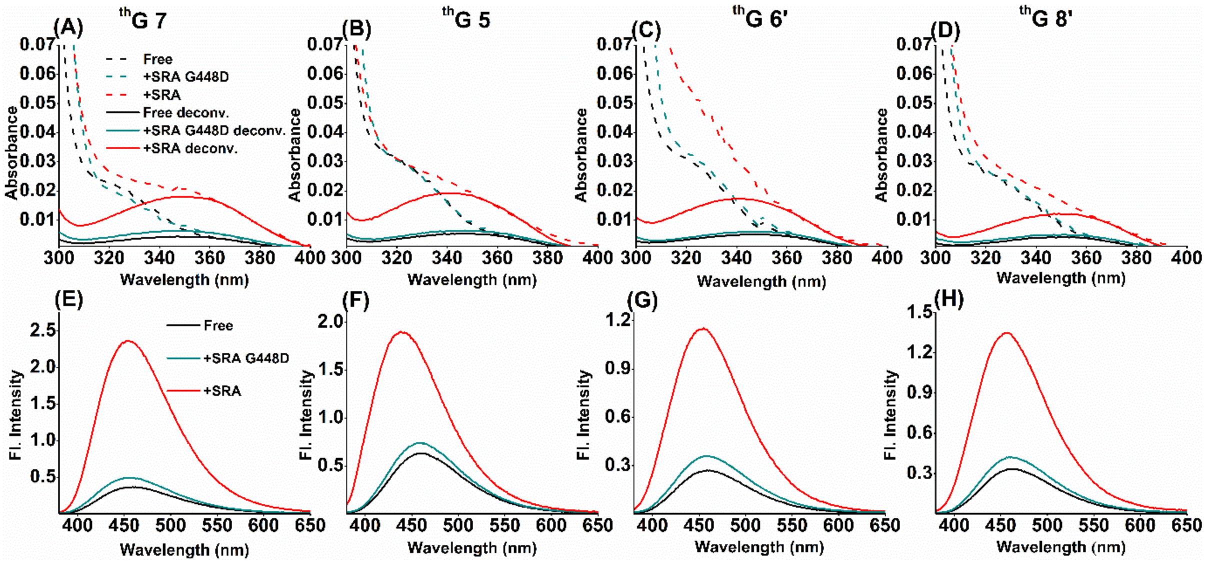

Figure S6: Steady-state absorption (A-D) and emission (E-H) spectra of the HM duplexes labelled at positions 7 (A,E), 5 (B, F), 6' (C, G) or 8' (D, H) in the absence and presence of SRA and SRA G448D. The experimental absorption spectra (A-D) of the free duplexes (black dotted lines), as well as those in the presence of SRA (red dotted lines) and SRA G448D (green dotted lines), have been deconvoluted using the corresponding excitation spectra at λ_{em} = 500 nm. The deconvoluted absorption bands (solid lines) describe the absorption of the nearly pure $\pi\pi^*$ excitation state localized on thG in the free duplexes and the complexes with proteins. The deconvoluted absorption bands of the thG excited states with charge transfer character that do not contribute to the emission of the labelled duplexes are omitted for clarity. The emission spectra (E-H) have been recorded at λ_{ex} = 350 nm. The concentrations of HM duplexes was 10 µM. The concentrations of SRA and SRA G448D were 12 µM and 20 µM, respectively. Buffer was as in Fig. S2.

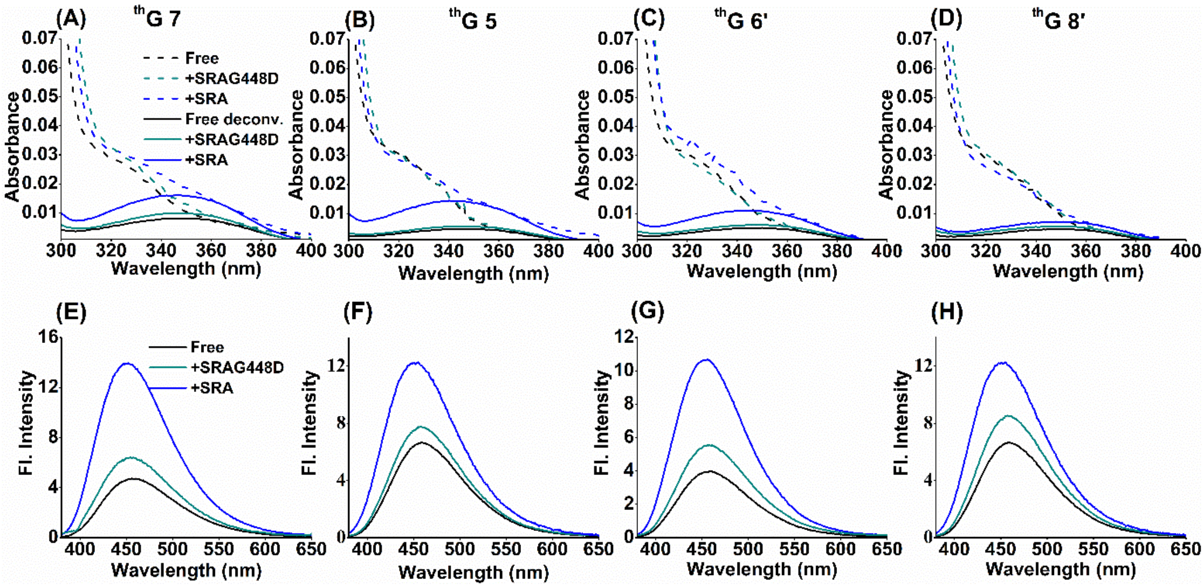

Figure S7: Steady-state absorption (A–D) and emission (E–H) spectra of the NM duplexes labelled at positions 7 (A, E), 5 (B, F), 6' (C, G) or 8' (D, H) in the absence and presence of SRA and SRA G448D. The experimental absorption spectra (A-D) of the free duplexes (black dotted lines), as well as those in the presence of SRA (red dotted lines) and SRA G448D mutant (green dotted lines), have been deconvoluted using the corresponding excitation spectra at λ_{em} = 500 nm. The deconvoluted absorption bands (solid lines) describe the absorption of the nearly pure ππ* excitation state localized on thG in the free duplexes and the complexes with proteins. The deconvoluted absorption bands of the thG excited states with charge transfer character that do not contribute to the emission of the labelled duplexes are omitted for clarity. The emission spectra (E–H) have been recorded at λ_{ex} = 350 nm. The concentrations of NM duplexes was 10 μM. The concentrations of SRA and SRA G448D were 12 μM and 20 μM, respectively. Buffer was as in Fig. S2.

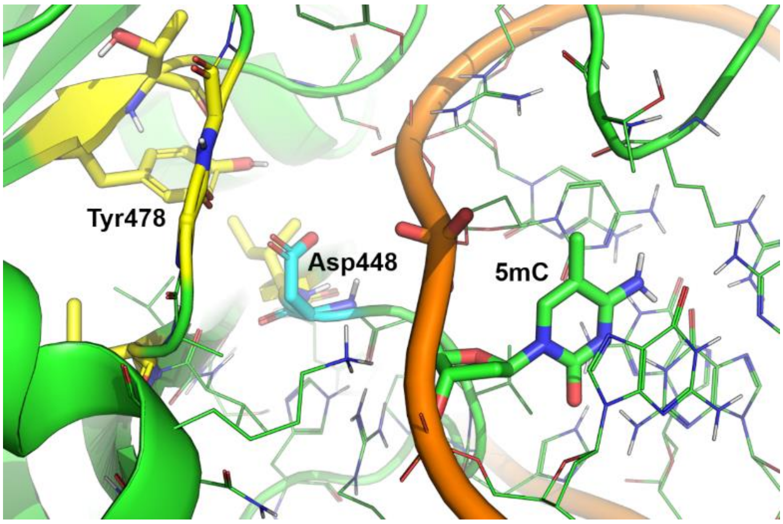

Figure S8. MD simulations of the complex between SRA G448D and HM DNA bearing 5mC at position 6. The larger Asp residue as compared to Gly residue in position 448 stably occupies the entrance of the SRA binding pocket, thus preventing base flipping. Accordingly, the 5mC base keeps its usual position in the duplex, and does not flip into the SRA binding pocket (in yellow). The representative frame extracted from the MD trajectory through cluster analysis is shown in cartoon and lines (only residues within 6 Å from 5mC are shown). Asp448 is coloured cyan; 5mC within the DNA duplex is shown as green sticks; Tyr478 is labelled as a reference residue in the SRA binding site.

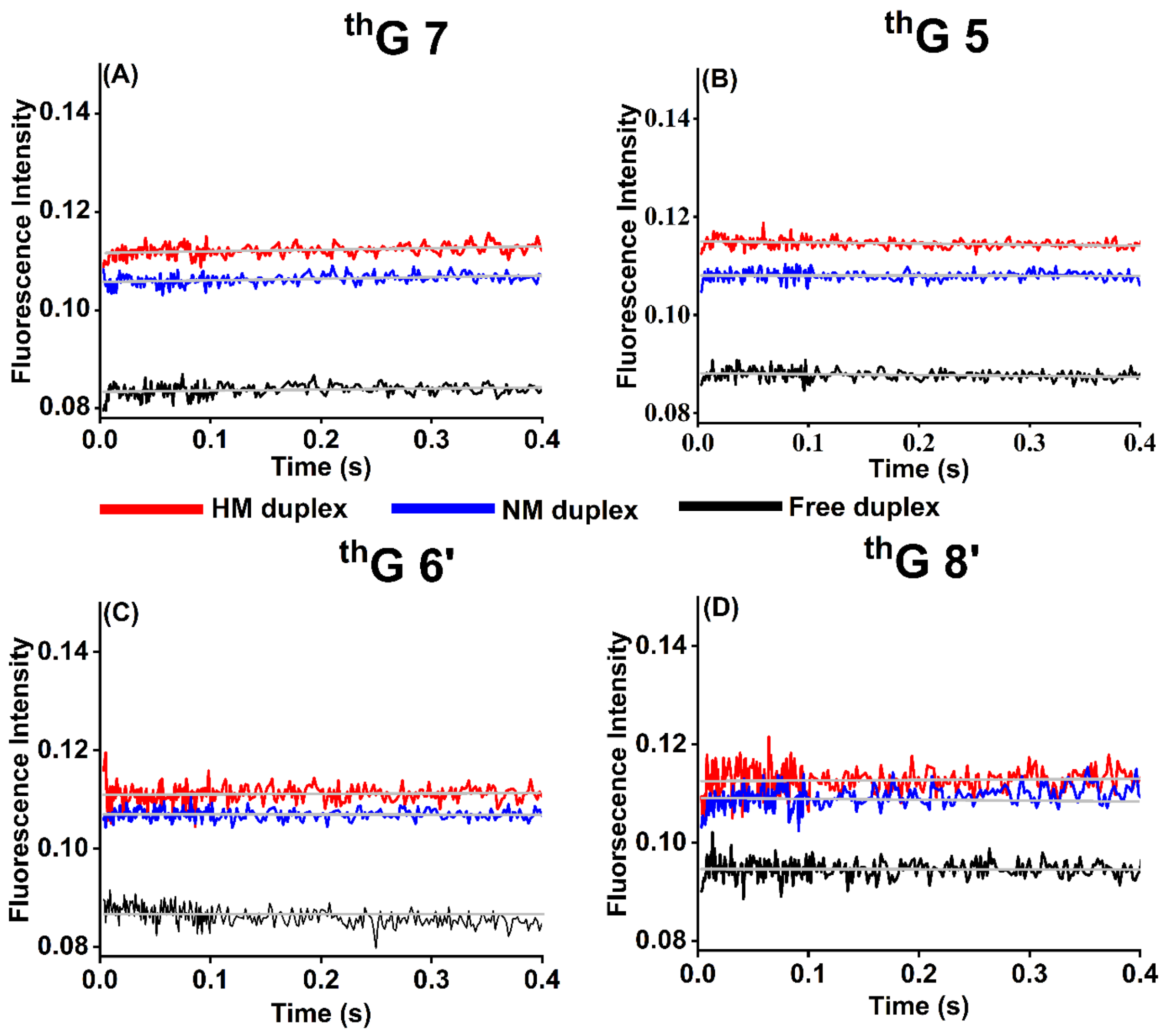

Figure S9: Stopped-flow kinetic traces of the interaction of SRA G448D with HM and NM duplexes labelled by thG at four different positions (7, 5, 6' and 8'). The black traces correspond to HM or NM duplexes mixed with the buffer. Red and blue traces correspond to the interaction of SRA G448D with HM and NM duplexes, respectively. The concentration of duplexes is 0.3 µM and the concentration of SRA G448D is 10 µM to ensure at least 80% complexation after mixing. Excitation wavelength is at 365 nm. Emission is collected above 420 nm with a long-pass filter. Data were collected with 2,000 points in the first 0.1 s and 9,000 points over the remaining 0.9 s. The data points have been further averaged by group of 4 (n = 4). Buffer is 20 mM PBS pH 7.5, 50 mM NaCl, 2.5 mM TCEP, 0.05% PEG 20000 at 20°C. No time resolvable phase is detected, indicating that thG fluorescence changes at all four positions occur exclusively within the dead time of the stopped-flow setup.

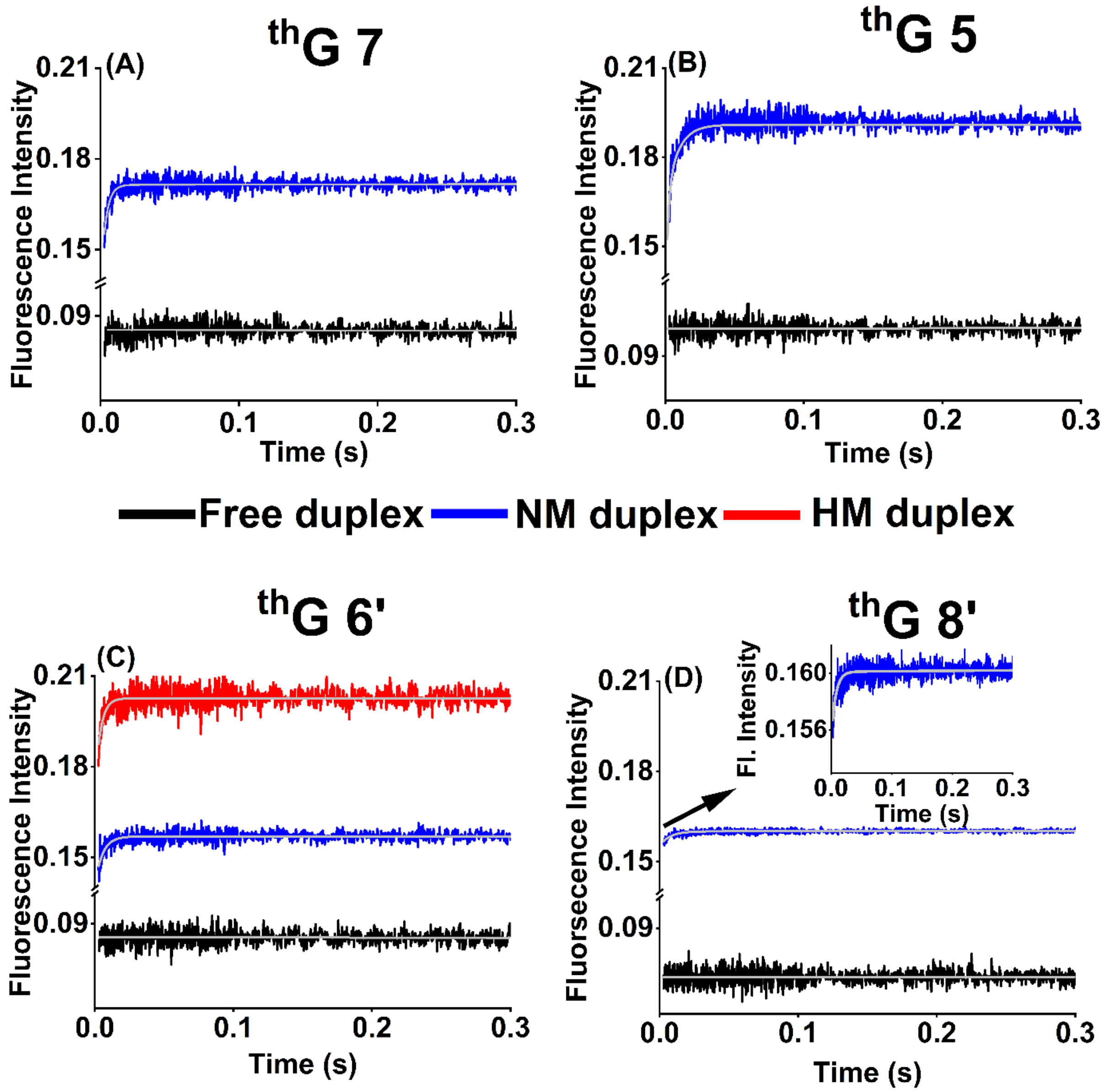

Figure S10: Stopped-flow kinetic traces highlighting the fast kinetic phase of the interaction of SRA with thG-labelled NM duplexes (A–D) and the HM duplex labelled by thG at position 6′ (C). This figure complements Figure 4 of the main manuscript. A Y-axis scale break has been applied to allow comparison with the corresponding free duplex signals. For the kinetic traces of the interaction of SRA with the NM duplexes (blue traces) and the HM duplex labelled at position 6′ (red trace), the solid grey lines correspond to their fits using equation (3) and the k_{obs} values given in Figure 4E-H. The inset in panel D provides a zoomed-in view to better visualize the fast phase of the interaction of SRA with the thG8′ NM duplex. The solid grey lines through the free duplex data (black lines) represent linear fits to the traces and define the I_0 value in Scheme 1.

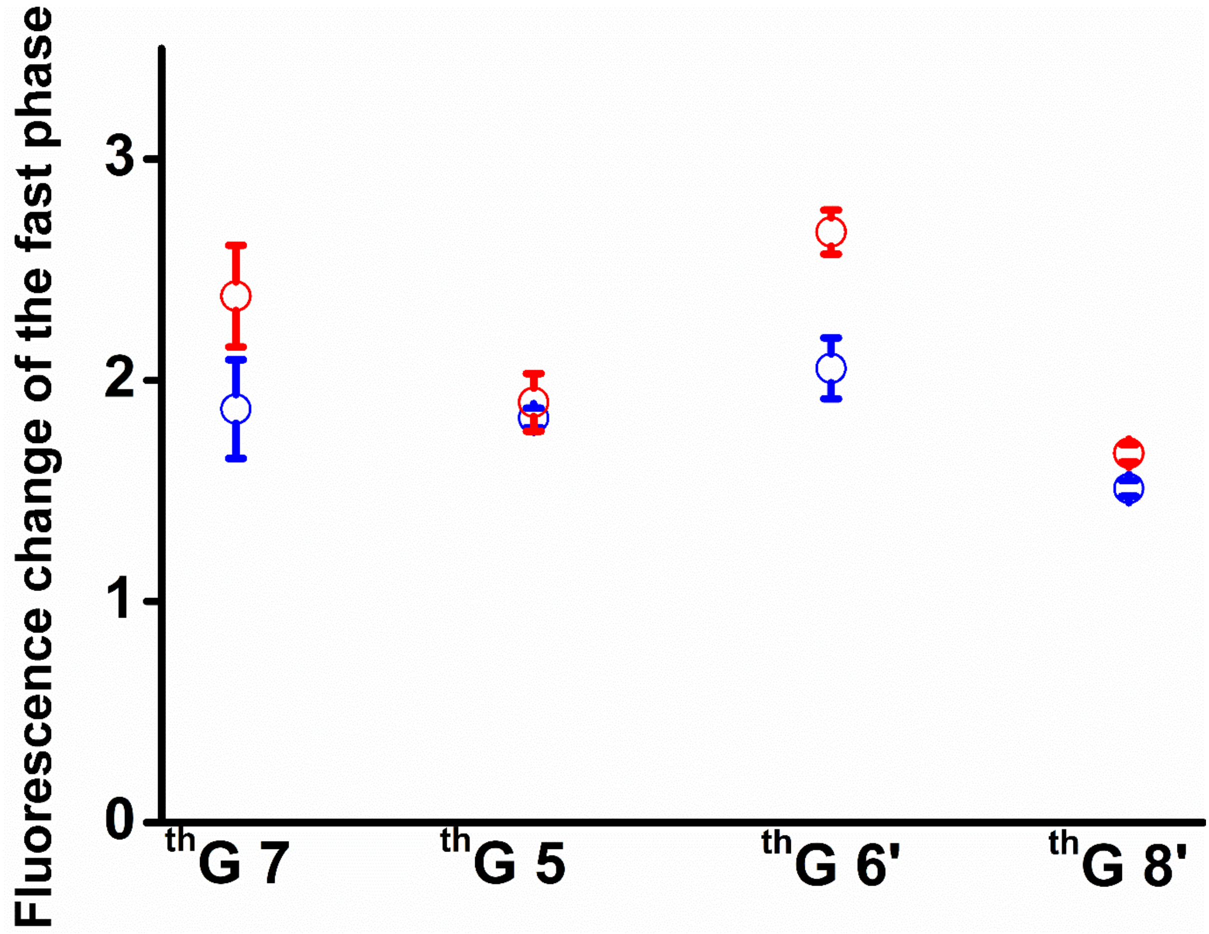

Figure S11: Comparison of the fluorescence change associated with the fast resolvable kinetic phase of SRA interaction with HM duplexes (red circles) with the fluorescence change observed for SRA interaction with NM duplexes (blue circles). The fluorescence change associated with the fast phase for HM duplexes has been calculated as $f_{HM}^{fast} = \frac{(I_{FHM}-I_i)*a+I_i}{I_i}$; where I_{FHM} is the final fluorescence intensity of the kinetic trace with the HM duplex at the plateau and I_i is the initial fluorescence intensity associated to the fast step, and a is the amplitude associated with the fast resolvable phase (red circles). The fluorescence change associated with the interaction of SRA with NM duplexes is calculated as $f_{NM} = \frac{I_{FNM}}{I_i}$ (blue circles), where I_{FNM} is the final fluorescence intensity of the kinetic trace with the NM duplex at the plateau. Each data point and associated error bar correspond respectively to the mean and standard deviation for at least two independent experiments.

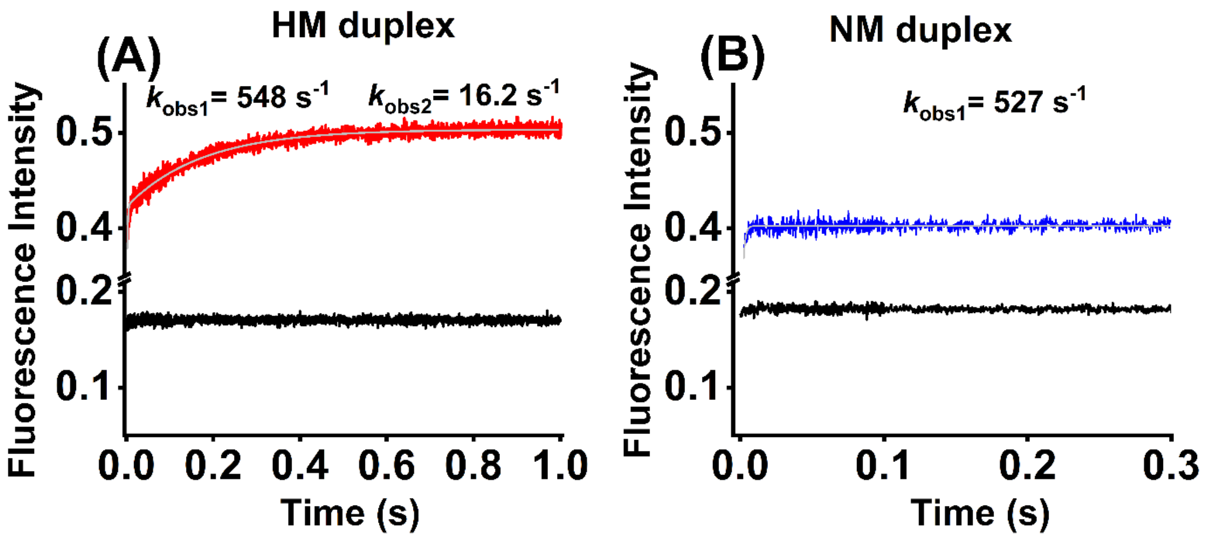

Figure S12: Stopped-flow kinetic profiles of SRA binding to HM (A) and NM (B) duplexes doubly labelled by thG at positions 7 and 8′. The black traces correspond to the free duplex mixed with the buffer. The grey line represents the fits to equation (3) of SRA interaction with the HM duplex (red trace) and the NM duplex (blue trace) using the k_{obs} values given in the two panels. Duplex concentration was 0.3 µM and SRA was used at 9 µM ([SRA]/[DNA] ratio = 30). Experiments were performed at 20°C in 20 mM PBS buffer (pH 7.5), 50 mM NaCl, 2.5 mM TCEP, and 0.05% PEG20000.

1

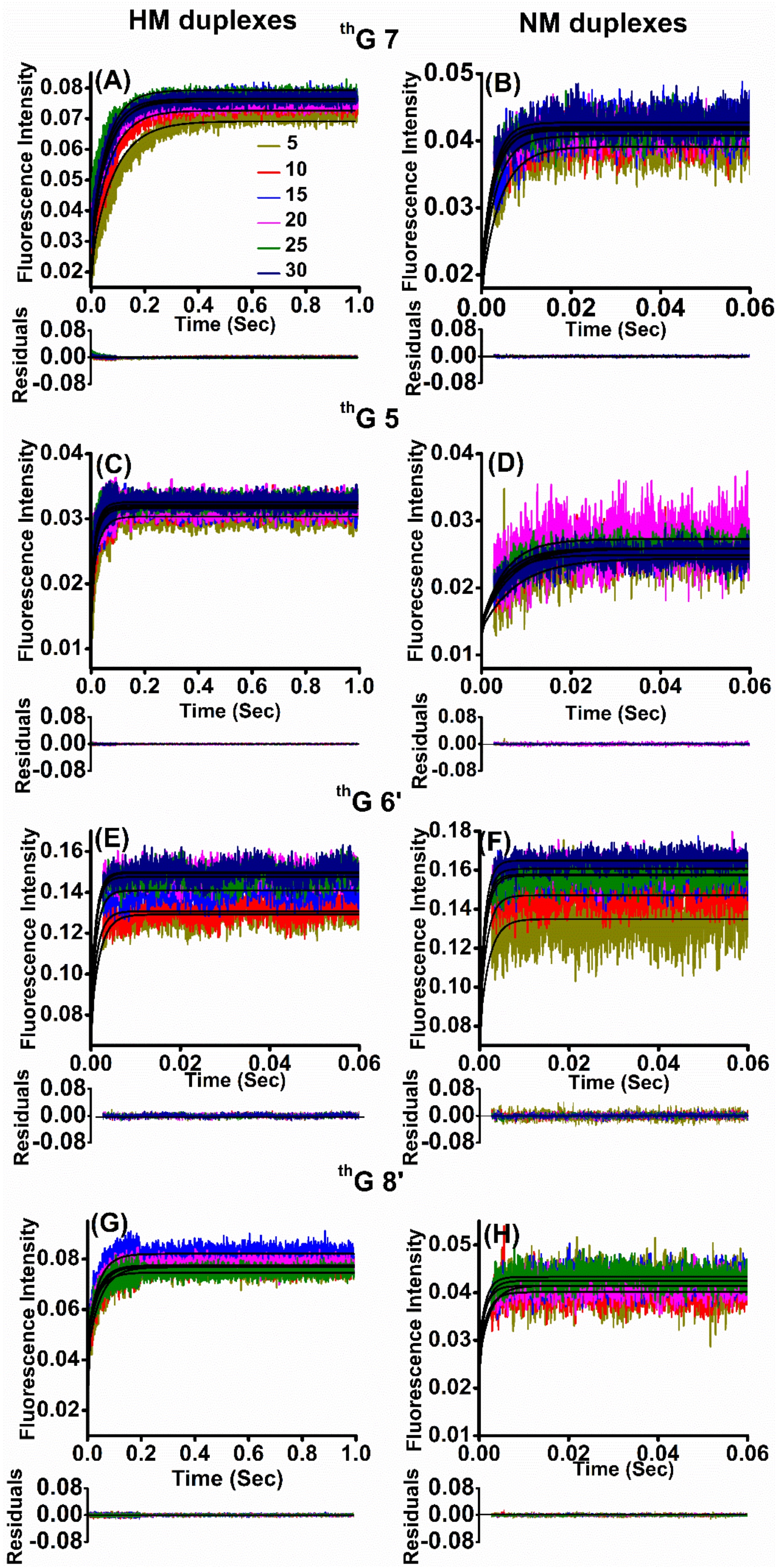

HM duplexes
NM duplexes
thG 7
thG 5
thG 6'
thG 8'
(A)
(B)
(C)
(D)
(E)
(F)
(G)
(H)
5
10
15
20
25
30
Fluorescence Intensity
Fluorecsence Intensity
Residuals
Time (Sec)

Figure S13: Global fit of the stopped-flow kinetic traces for the interaction of SRA with HM and NM duplexes labelled by thG at position 7 (A, B), 5 (C, D), 6' (E, F) and 8' (G, H). The duplexes at 0.3 µM were mixed with SRA at the final SRA/DNA concentration ratios indicated in the upper left panel. Buffer conditions were as in Figure 4. In each panel, the kinetic traces were globally fitted (black lines) by using the Dynafit software and the values of the kinetic rate constants given in Figure 6. The residuals of the fits are displayed below each trace to assess the goodness of fit.

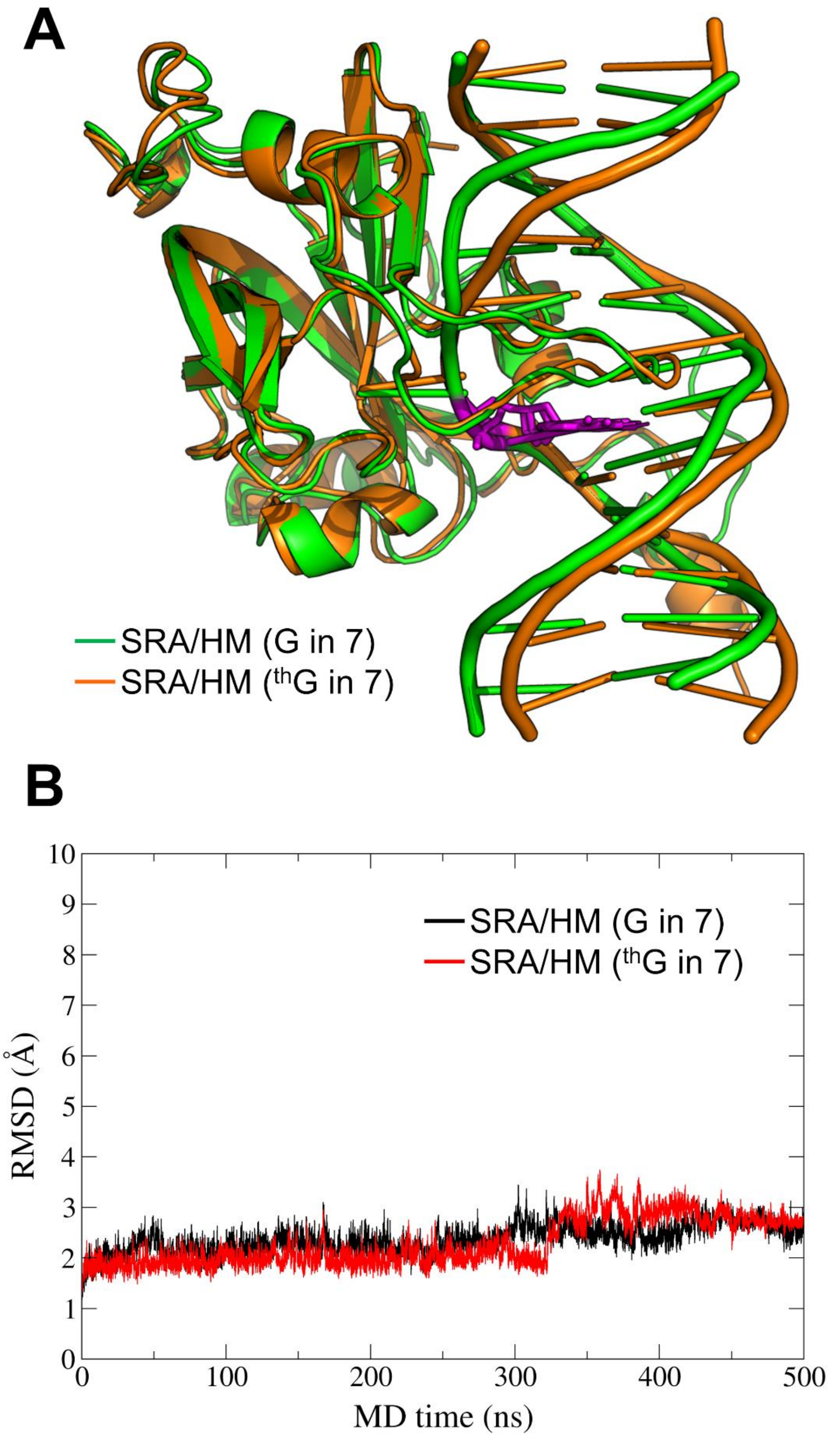

Figure S14: (A) Structural overlap of the SRA/HM complexes containing G or thG7 in position 7 (magenta sticks). (B) Plot of the root-mean-square deviation (RMSD) of both SRA/HM complexes throughout the molecular dynamics (MD) simulation time.

Additional discussion of the spectroscopic data and QM results

The aim of this chapter is to provide an explanation for the interested readers about the unusual origin of the fluorescence changes of thG-labelled duplexes associated to SRA-induced base flipping and initially described in (1). Indeed, while fluorescence changes are generally associated to changes in the probe environment or excited-state reactions that modify the non-radiative pathways, the fluorescence changes of thG-labelled duplexes in Figure 3 are almost entirely related to the changes in absorbance and more specifically to those of the lowest energy bright state of thG, as highlighted in the panels A-D of Figures S6 and S7 (spectra in solid lines). This lowest energy excited state is localized on the thG base (thG*, Table S1) and is responsible of the emission of the labelled duplexes. This excited state is of the same kind as that of the free thG base in water, but differs from it by its energy -as shown by the 15 nm red-shift of its absorption maximum- and its oscillator strength (absorption coefficient). The change in oscillator strength in respect to the free probe depends on the nature of the flanking bases, as illustrated in our previous paper (1). Indeed, the observed hypochromicity as compared to the free thG base in water was considerably more pronounced when thG was flanked in 3' with a C residue as compared to an A residue. In fact, both the strong shift in absorption maximum and the strong hypochromicity suggest that, as in the case of natural nucleosides in DNA, thG is electronically coupled with its close neighbours, forming a new entity with collective behavior and spectroscopic properties different from those of free thG. Moreover, as a result of the highly dynamic behavior of DNA duplexes, there is likely a collection of close conformations with slight changes in distances and orientations between thG and its neighbors, providing a continuum of electronically coupled entities. Therefore, the nature of the flanking nucleobases, the dynamics of the duplexes and the energy of light are expected to control the populations and the absorption coefficient of the red-shifted thG* band and the blue-shifted charge transfer state in Figures S6 and S7.

Concerning the fluorescence properties, the similarity in fluorescence quantum yields and lifetimes for different labelled sequences (Figure 7 and Table 1 in (1)) indicates that the nature of the flanking residues of thG plays only a limited role on the values of these fluorescence parameters. The next question then is: does the microenvironment of thG within DNA duplexes have any impact on these parameters? Since both fluorescence lifetimes (~ 10 ns) and quantum yields (~0.25) values are only about half of those of free thG in water, the answer to this question is clearly yes. Part of this effect can be related to

the aforementioned electronic coupling of thG with its flanking residues, making possible, for instance, partial charge transfer reactions that decrease the quantum yield and lifetime values. An additional explanation is related to the polarity of thG microenvironment within the duplex, which is smaller than in water, as can be seen from the ~10 nm blue shift of its emission maximum. The effect of the polarity is well illustrated by the data of free thG in methanol and ethanol, where a comparable blue shift in the emission maximum is observed. Since both alcohols are assumed to mimic the polarity within DNA duplexes and since both of them induce a ~ 25% decrease in the fluorescence quantum yield and lifetime as compared to water, this can partly explain the data of thG in DNA duplexes.

When SRA is added and flips 5mC, a loss of stacking with one of its flanking residues (either at the 3' or 5' end) is predicted for thG at position 5, 7, and 6'. However, the stacking with the residue at other end is preserved and thG remains within the duplex in the same environment. As a result of the partial loss of stacking, the electronic coupling of thG with its neighbors is smaller, which explains the increase in the absorption coefficient, also evidenced by QM calculations (see below). The population of thG molecules able to occupy the thG* state is likely also increased. In contrast, since the protein has only limited contact with nucleosides inside the duplex and does not deeply impact the environment of thG within the duplex (2), the fluorescence quantum yield and lifetime only change marginally on SRA binding.

For the QM results, it is important to highlight that a full assessment of all the possible factors (radiative and non radiative deactivation paths, intersystem crossing, vibronic couplings) modulating the spectral dynamics of a probe included in a duplex, even more when interacting with a protein, is, still out of reach for any theoretical analysis. It would be indeed necessary to simulate the excited state dynamics of a fluctuating system containing thousands of atoms, on a time scale ranging from the femtosecond to the millisecond or even beyond. On the other hand, keeping in mind the limitations of any analysis based on the static description of the excited states of a few oligonucleotide structures, and discarding the possible role of the protein, QM calculations can give very useful atomistic insights on the main chemical/physical processes into play, as shown, inter alia, by many of our previous studies on DNA photophysics and photochemistry (3-6).

Thus, in Table 1 we report a concise description of the lowest energy excited states of four representative oligonucleotide structures, issuing from the MD simulations, after optimizing their ground state minimum. An oligonucleotide at room temperature is a fluctuating system, where multiple local minima are in equilibrium. On the other hand, based on our experience on DNA fragments, also when containing a fluorescent probe like thG, the simulated absorption and circular dichroism spectra of the minimum well reproduce their experimental counterpart, indicating that the DNA fragments are fairly representative structures of the oligonucleotide in solution.

In all the duplex fragments considered, the lowest energy excited state is localized on the thG base (thG*). However, the energy and the oscillator strength of this excited state change in the different structures. In particular, in the non flipped structure, where thG is stacked with two bases (5mC and C), we observe that the oscillator strength is 25% smaller than in the flipped structures, where 5mC is not anymore stacked to thG. This result is well consistent with the experimental increase of the absorbance induced by the 5mC base flipping. Another interesting feature emerging from the analysis of the absorption spectra is the presence of an excited state with significant charge transfer (CT) character involving thG and its stacked C8 partner. This excited state, which can act as effective quencher of the thG fluorescence, is relatively more stable (energy gap of only ~0.65 eV from thG*) in the non flipped structure, constituting another element that explains the increase of the fluorescence upon flipping. It is thus clear that the analysis of the lowest absorption band, as we did in (1), provides very useful hints to interpret the experimental spectra. On the other hand, considering that the experimental analysis focuses on the fluorescence data, and to avoid the risk of overinterpreting the results concerning a single, yet, representative structure, in this study we decide to base our analysis mainly on the features of the most relevant excited state minima of the four structures, which is reported in the main text.

Table S1 Vertical Absorption energies (VAE), oscillator strength and schematic description of the five lowest energy excited states computed for the 5mCthGC or thGC fragments of different representative structures. PCM/TD-M052X/6-31G(d) calculations in water.

GS minimum	Energy (oscillator strength)	Description
Unflipped structure		
S_1<br>after geometry optimization	4.1384 (0.1471)<br>3.2063 (0.1740) thG*-min	thG*
S_2<br>after geometry optimization	4.7394 (0.0176)<br>2.9845 (0.0059) thGC-CT-min	thG$\rightarrow$C8
S_3	5.1057 (0.1682)	5mC* + thG$\rightarrow$5mC
S_4	5.1425 (0.0160)	G8'$\rightarrow$C7'
S_5	5.1456 (0.0069)	thG$\rightarrow$5mC
Flipped		
S_1<br>after geometry optimization	4.2306 (0.1915)<br>3.2814 (0.2062) thG*-min	thG*
S_2<br>after geometry optimization	4.9870 (0.0450)<br>3.2432 (0.0161) thGC-CT-min	thG$\rightarrow$C8 +C8*
S_3	5.0778 (0.0112)	G8'$\rightarrow$C7'
S_4	5.1829 (0.0688)	C7'*
Flipped frame (G6' close) 250 ns		
S_1<br>after geometry optimization	4.2717 (0.1922)<br>thG*/ thGC-CT min 3.098 (0.1246)<br>thG*-min 3.1870 (0.2054)	thG*
S_2<br>after geometry optimization	4.9659 (0.0038)<br>decay to thG*	thG$\rightarrow$C8
S_3	4.9891 (0.0100)	G8'$\rightarrow$C7'
S_4	5.1084 (0.0202)	G6'$\rightarrow$thG
Flipped frame (G6' far)400 ns		
S_1<br>after geometry optimization	4.1030 (0.1820)<br>3.1268(0.1915) thG*-min	thG*
S_2<br>after geometry optimization	4.9137 (0.0451)<br>decay to thG*-min	thG$\rightarrow$C8
S_3	4.9979(0.0279)	G8'$\rightarrow$C7'
S_4	5.1390(0.0147)	G6'$\rightarrow$C7' mainly

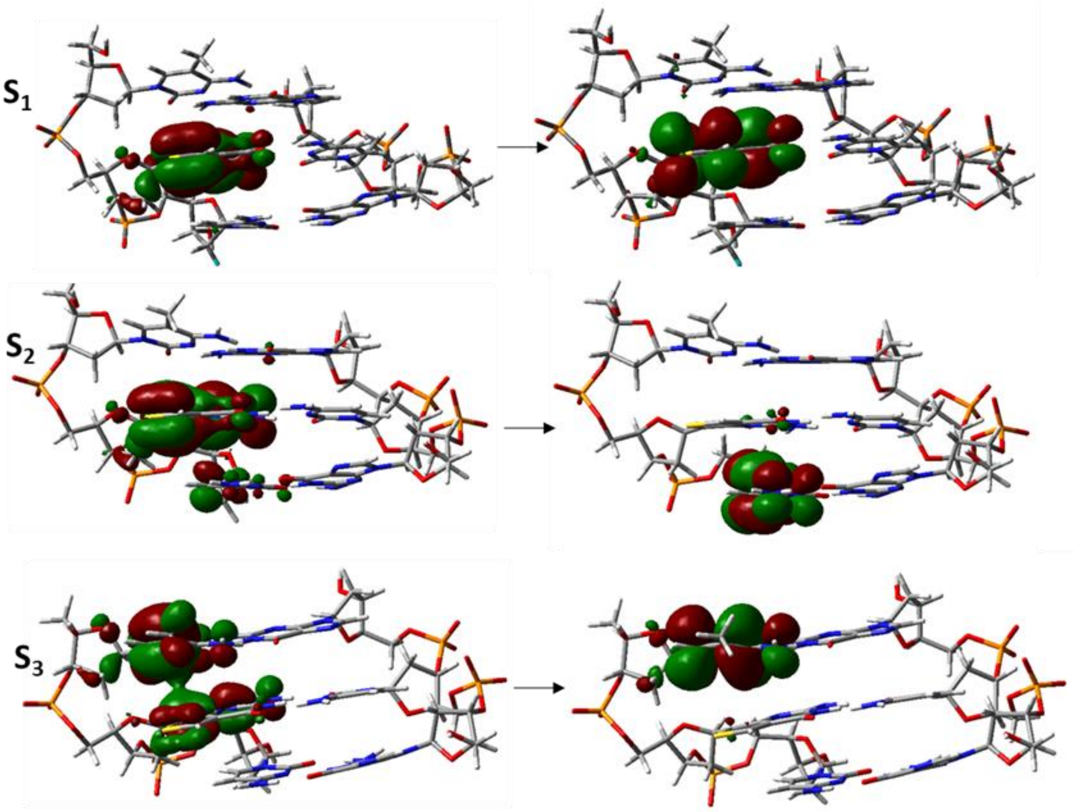

Figure S15: Natural transition orbitals (NTO) associated to the three lowest energy excited states for the 5mCthGC/GCG fragment of the free duplex, according to PCM/TD-M052X/6-31G(d) calculations.

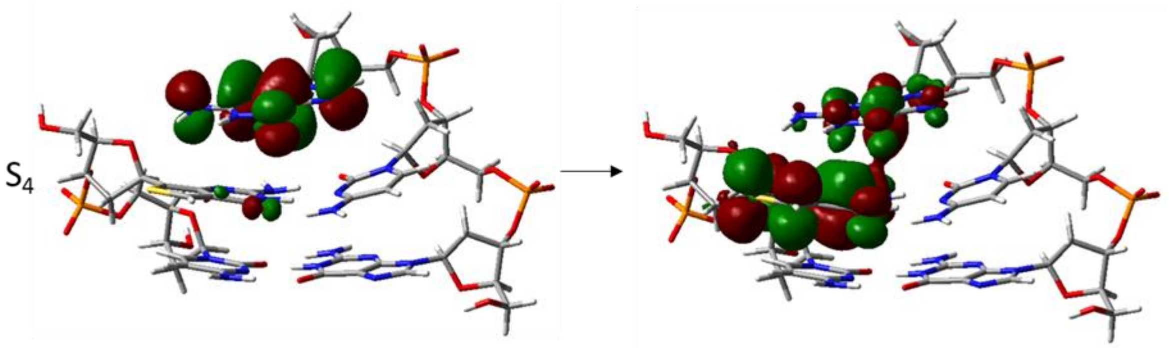

Figure S16: Natural transition orbitals (NTO) associated to the fourth lowest energy excited state for 'G6' close', the thGC/CG fragment of the MD simulation frame at 250 ns, according to PCM/TD-M052X/6-31G(d) calculations.

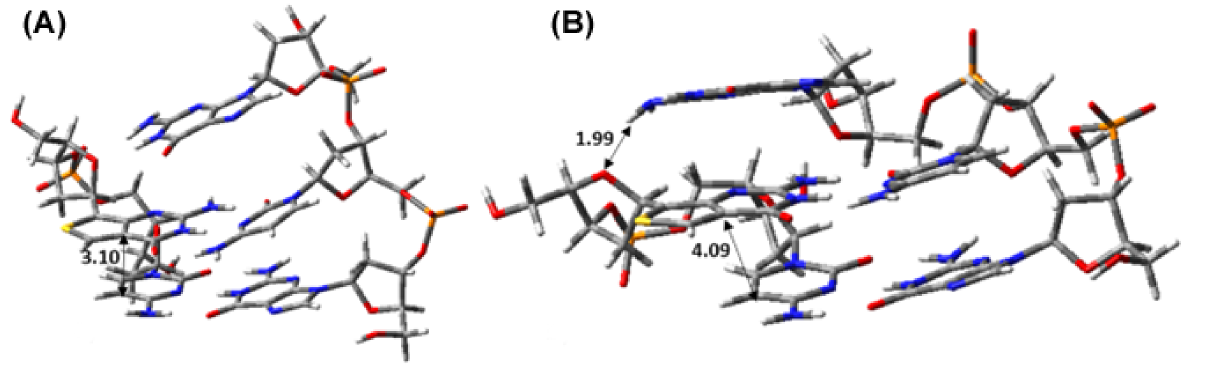

Figure S17: Schematic drawing of a) the minimum geometry of the excited minimum with mixed thG*/thGC-CT character and b) the 'pure' thG*-min optimized for 'G6' close', the thGC/CG fragment of the frame at 250 ns, by PCM/TD-M052X/6-31G(d) calculations.

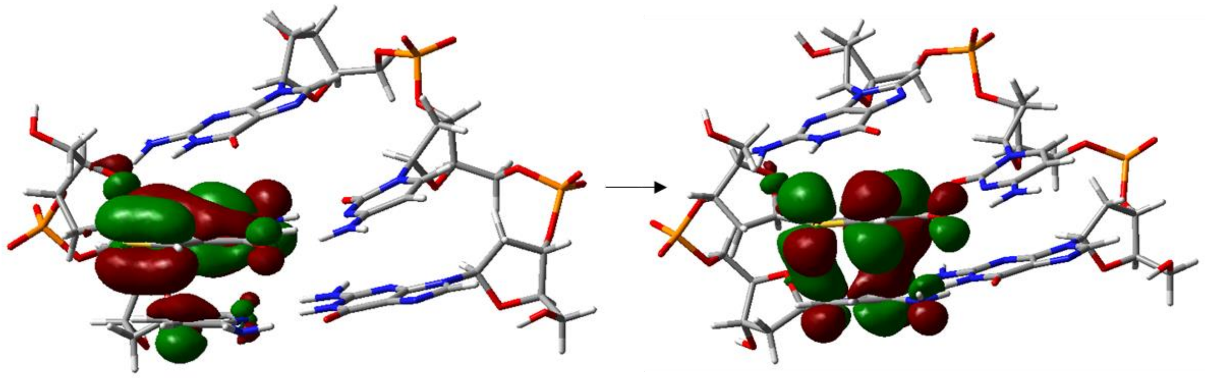

Figure S18: Schematic drawing of the natural transition orbitals (NTO) associated to the excited minimum with mixed thG*/thGC-CT character optimized for ‘G6' close’, the thGC/CG fragment of the MD simulation frame at 250 ns, by PCM/TD-M052X/6-31G(d) calculations.

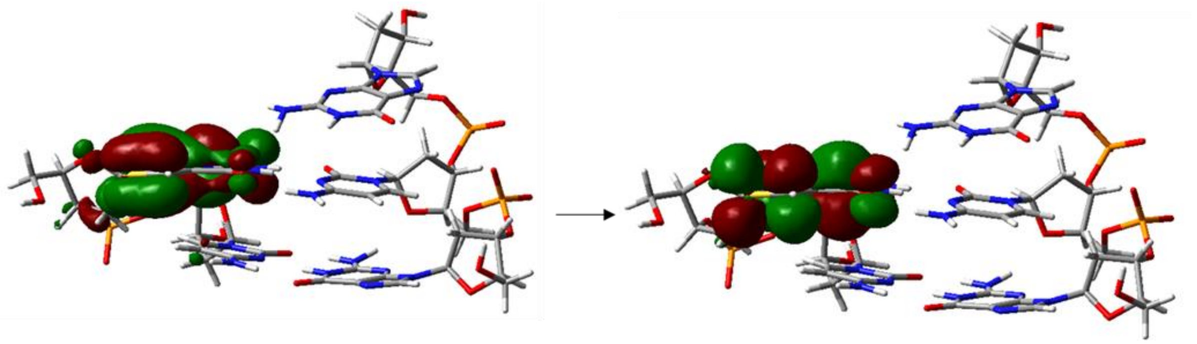

Figure S19: Schematic drawing of the natural transition orbitals (NTO) associated to the thG* minimum optimized for ‘G6' far’, the thGC/CG fragment of the MD simulation frame at 400 ns, by PCM/TD-M052X/6-31G(d) calculations.

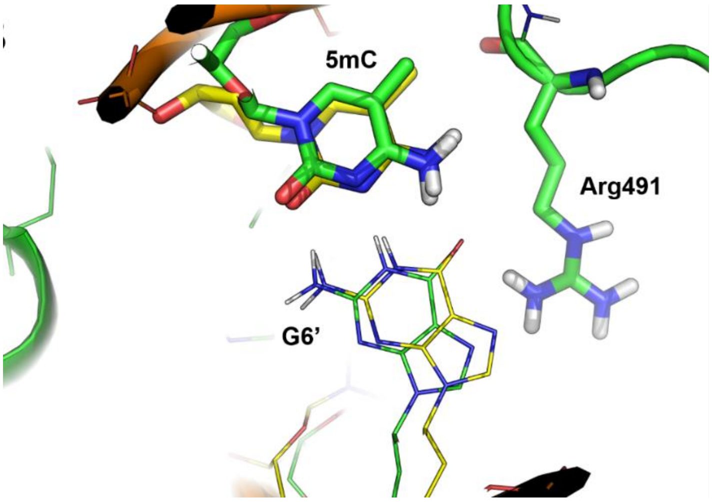

Figure S20: Comparison between the free duplex (yellow) and the HM duplex/SRA G448D complex (green) showing a displacement of the G6' residue in the complex as a result of the insertion of the Arg 491 residue. The mean distance between G6' and thG7 is 6.85 Å, as compared to 6.2 Å in the free duplex (calculated between G6' and G7).

Reference

1. Ciaco, S., Gavvala, K., Greiner, V., Mazzoleni, V., Didier, P., Ruff, M., Martinez-Fernandez, L., Improta, R. and Mély, Y. (2022) Thienoguanosine brightness in DNA duplexes is governed by the localization of its ππ* excitation in the lowest energy absorption band. *Methods Appl. Fluoresc.*, **10**, 035003.
2. Avvakumov, G.V., Walker, J.R., Xue, S., Li, Y., Duan, S., Bronner, C., Arrowsmith, C.H. and Dhe-Paganon, S. (2008) Structural basis for recognition of hemi-methylated DNA by the SRA domain of human UHRF1. *Nature*, **455**, 822-825.
3. Improta, R., Santoro, F. and Blancafort, L. (2016) Quantum mechanical studies on the photophysics and the photochemistry of nucleic acids and nucleobases. *Chem. Rev.*, **116**, 3540-3593.
4. Gustavsson, T., Improta, R. and Markovitsi, D. (2010) DNA/RNA: building blocks of life under UV irradiation. *J. Phys. Chem. Lett.*, **1**, 2025-2030.
5. Martínez Fernández, L., Santoro, F. and Improta, R. (2022) Nucleic acids as a playground for the computational study of the photophysics and photochemistry of multichromophore assemblies. *Acc. Chem. Res.*, **55**, 2077-2087.
6. Martínez-Fernández, L., Pepino, A., Segarra-Martí, J., Jovaisaite, J., Vaya, I., Nenov, A., Markovitsi, D., Gustavsson, T., Banyasz, A. and Garavelli, M. (2017) Photophysics of deoxycytidine and 5-methyldeoxycytidine in solution: a comprehensive picture by quantum mechanical calculations and femtosecond fluorescence spectroscopy. *J. Am. Chem. Soc.*, **139**, 7780-7791.
